# Incidence and Prevalence of Reported Euthanasia Cases in Belgium, 2002 to 2023

**DOI:** 10.1001/jamanetworkopen.2025.6841

**Published:** 2025-04-23

**Authors:** Jacques Wels, Natasia Hamarat

**Affiliations:** 1 MRC (Medical Research Council) Unit for Lifelong Health and Ageing, University College London, London, United Kingdom; 2 Health & Society Research Unit, Université Libre de Bruxelles, Brussels, Belgium; 3 Centre de Droit Public et Social, Université Libre de Bruxelles, Brussels, Belgium

## Abstract

**Importance:**

Reported cases of assisted dying have increased in countries with such legislation. In Belgium, where euthanasia was legalized in mid-2002, cases rose from 236 in 2003 to 3423 in 2023. Most previous studies have focused on occurrence rates.

**Objective:**

To examine the magnitude of the increase in euthanasia cases and its association with demographic changes observed during the study period.

**Design, Setting, and Participants:**

This cross-sectional study analyzed complete data from the Belgian Federal Commission for the Control and Evaluation of Euthanasia (FCCEE) from September 1, 2002, to December 31, 2023, and adjusted the model for demographic composition and change by gender, age group, and region using data from the Belgian Office for Statistics. All cases of euthanasia reported to the FCCEE during the selected period were included.

**Exposure:**

Euthanasia reported to the FCCEE.

**Main Outcome and Measures:**

Poisson regression with and without a demographic offset was used to provide the rate ratios (RRs) and the prevalence rates (PRs) for euthanasia. The RRs were calculated by age, gender, region, and euthanasia characteristics. Estimates used a model including demographic offsets to calculate PRs and explore interactions across subcategories.

**Results:**

During the selected period, 33 647 cases of euthanasia were reported (50.23% male; 84.74% 60 years or older); analyses focused on 33 580 valid cases. The yearly RR was 1.07 (95% CI, 1.07-1.07), while the yearly PR was 1.05 (95% CI, 1.05-1.06), indicating that demographic changes were associated with the observed increase. The PR for euthanasia among males relative to females was overall higher (PR, 1.36; 95% CI, 1.33-1.39) but has decreased slightly (PR, 0.99; 95% CI, 0.99-1.00). Cases citing multimorbidity increased relative to those citing tumors (PR, 1.03; 95% CI, 1.02-1.04), whereas cases related to psychiatric disorders and deaths in care homes did not show significant increases. Higher prevalence was observed in the Flemish region relative to Wallonia (PR, 1.51; 95% CI, 1.47-1.55), but the gap has narrowed over the years.

**Conclusions and Relevance:**

This study found that a substantial part of the increase in euthanasia cases was attributable to demographic changes. Early increases were mainly due to the regulatory onset, while recent trends reflect a growing influence of demographic factors and regional adjustments. These findings suggest that considering demographic shifts is essential, and long-term trends should be monitored.

## Key Points

**Question:**

What factors are associated with patterns in euthanasia incidence and prevalence in Belgium?

**Findings:**

In this cross-sectional analysis of all 33 580 valid euthanasia cases since 2003, with adjustments for demographics, approximately one-fourth of the increase in cases could be accounted for by demographic changes. There was harmonization across genders and regions, while cases involving psychiatric and cognitive conditions remained stable.

**Meaning:**

These findings suggest that the euthanasia safeguards implemented in Belgium appear effective, as patterns in euthanasia reflect the initial effects of legislation, harmonization across subgroups, and the impact of an aging population.

## Teaser

This cross-sectional study examines patterns in euthanasia incidence and prevalence in Belgium since its legalization in 2002.

## Introduction

Cases of assisted dying have steadily risen in countries with legalized euthanasia or assisted suicide. In the Netherlands, reported cases increased from 1933 in 2005 to 6361 in 2019, representing 4.4% of all deaths in 2017, up from 1.9% in 1990.^1^ Switzerland saw a similar trend, with requests tripling for older women and doubling for men between 1991 and 2008.^2^ This growth is attributed to more favorable attitudes toward assisted dying,^3^ and more countries are implementing or debating assisted dying policies.^4^

In Belgium, reported euthanasia—the form of assisted dying where a medical practitioner actively ends a patient’s life at their explicit request—increased from 236 cases in 2003 to 2700 cases in 2021, accounting for 2.4% of all deaths.^5,6^ The country legalized euthanasia in 2002, allowing competent adults in a medically hopeless condition who are suffering unbearably from a serious disorder to request euthanasia, provided it is voluntary, in writing, and approved by independent physicians.^7^ In 2014, the law was extended to minors, involving only a few cases.^8^ The Federal Commission for the Control and Evaluation of Euthanasia (FCCEE) ensures compliance and transparency.^9^

A common concern is that allowing voluntary active euthanasia for some specific conditions would result in a so-called slippery slope,^10^ suggesting that once euthanasia is permitted for specific conditions, it may lead to broader, less ethically acceptable practices, such as for nonterminal or psychiatric conditions.^11,12^ This concern emphasizes the need for rigorous safeguards, particularly in cases involving psychiatric disorders.^13^ Additionally, critics argue that socioeconomically vulnerable groups,^14^ including those in underresourced health care settings^15^ or deprived care facilities,^12^ could be disproportionately affected, raising concerns about potential coercion. Euthanasia, in this configuration, would not be efficiently monitored and controlled and might lead to error or abuse of the rights of vulnerable patients.^16^ However, studies focusing on the slippery slope assumption rarely focus on data,^17^ and empirical investigations do not attest the existence of a slippery slope in the Netherlands^18^ or Oregon.^19^

Physical suffering without prospects of improvement is the most common reason given for granting euthanasia,^20^ and euthanasia is mostly linked to chronic or terminal physical conditions, with a large share due to cancers in terminal phase.^21^ Euthanasia for nonterminal illness is allowed in Belgium, but the issue remains controversial and highly debated.^22^ Between 2002 and 2021, reported euthanasia for patients with psychiatric disorders concerned 370 cases, 1.4% of the total number of euthanasia cases.^23,24^

A common pitfall in addressing euthanasia trends is overlooking demographic changes. Aging populations, regional distribution, and the higher proportion of women in older age groups may impact euthanasia incidence. Older adults may experience higher rates due to terminal illnesses, while regional differences may reflect access to euthanasia services or cultural attitudes.^25,26^ Some studies explored euthanasia incidence among specific subgroups. In Belgium, administrative data show a balanced gender distribution, with women representing 49.6% of euthanasia cases in 2020,^27^ and similar euthanasia rates across genders. Women are overrepresented in psychiatric cases, although these represent a small fraction of total cases.^28,29^ Regional differences also exist. In the Netherlands, unexplained geographical variations were observed, influenced by factors such as age, religion, political orientation, income, self-perceived health, and availability of volunteers providing informal care, though much remains unexplained.^1^ In Belgium, euthanasia is more common in the Flemish region,^30^ with most research focused on Flanders.^31,32^

As several nations are currently debating the potential introduction of assisted dying bills, with cautionary perspectives often dominating the discourse over calls for legislation,^33,34^ more research is needed to thoroughly understand euthanasia prevalence in countries that have implemented it.^4^ This study analyses Belgian administrative data on euthanasia (2002-2023) to (1) assess prevalence by age, gender, region, and health condition; (2) compare these figures with population trends; and (3) identify subgroup trends.

## Methods

### Data

We use data routinely collected by the FCCEE, derived from individual reports that medical practitioners are legally required to submit for each case. These data are fully anonymized and encompass all reported euthanasia cases since 2002. The FCCEE granted ethical approval on May 14, 2024. Consent to participate was waived by the FCCEE in accordance with Belgian regulations. Additionally, we used open population data provided by the Belgian Office for Statistics to calculate population sizes by area of residence, gender, and age. This study follows the Strengthening the Reporting of Observational Studies in Epidemiology (STROBE) reporting guideline for cross-sectional studies.

The dataset included 33 647 cases, representing all reported cases in Belgium between September 1, 2002, and December 31, 2023. We excluded data from 2002 in our empirical models because the law was implemented in mid-2002, which resulted in low case numbers for that year (n = 24). Additionally, 43 cases were removed due to incomplete information. Missing data were not imputed, given their small proportion (0.1% of the total) and limited available information. The final sample included 33 580 cases.

### Euthanasia Variables

The study focuses on the following 9 variables available within the FCCEE dataset.

Reasons for euthanasia: The FCCEE identifies 12 possible medical conditions that can justify euthanasia. In this study, we classified reasons under 7 categories: (1) cancer and tumors (reference group), (2) multimorbidity, (3) nervous system diseases, (4) specific diseases, (5) psychiatric disorders, (6) cognitive disorders, and (7) others. Specific diseases include diseases of the respiratory system; diseases of the circulatory system; diseases of the genitourinary system; diseases of the digestive system; hematological disorders; endocrine, nutritional, and metabolic diseases; diseases of the eye and its adnexa; diseases of the ear and mastoid process; diseases of the musculoskeletal system, muscles, and connective tissue; and diseases of the skin and subcutaneous tissue. Other reasons included symptoms, signs, and abnormal clinical and laboratory findings not elsewhere classified; traumatic injuries; poisoning; and certain other consequences of external causes. We maintained the FCCEE distinction between psychiatric and cognitive disorders due to their different clinical profiles.
Age group: The FCCEE data include birth and death dates, but to preserve anonymity, we used 8 postcalculated age groups: 15 to 29, 30 to 39, 40 to 49, 50 to 59 (reference group), 60 to 69, 70 to 79, 80 to 89, and 90 years or older.
Gender: As reported by the medical practitioner, gender was recorded as male or female (reference group).
Language: Belgium’s federal structure includes 3 regions (Wallonia, Flanders, and Brussels). While the patient’s place of residence was not consistently collected by the FCCEE until recently, the language (Dutch or French) used by the reporting medical practitioner is systematically included. This allowed us to impute regional differences by distinguishing euthanasia cases reported in Dutch or French (reference group).
Year: The dataset records the year the euthanasia was carried out, ranging from 2002 to 2023. We excluded 2002 because the onset of the regulation occurred in mid-2002.
Basis for euthanasia: The dataset distinguishes between euthanasia requests made in advance (advanced request) or at the time of need (actual; reference group).
Type of suffering: The dataset includes information on the type of unbearable suffering reported by the practitioner, categorized as physical suffering (reference group), mental suffering, or both.
Term of death: The dataset records whether death was expected to occur within a year or over a longer period (reference group), as reported by the physician.
Place of death: The dataset distinguishes several types of places where the euthanasia was performed, including home (reference group), hospital, care home, palliative care, and other.

### Population Variables

We generated population figures based on demographic data retrieved from the Belgian Office for Statistics. This included information on the total population as of January 1 for each selected year, broken down by age group, sex, and region of residence. We chose to use population figures instead of the number of deaths (or nonviolent deaths), as done in previous studies,^1,20,24,35,36^ because a nonnegligible share of euthanasia is performed for patients not expected to die in the foreseeable future (for example, 14.4% of all cases in 2020 to 2021).^27^ The figures are calculated for each line of euthanasia counts by year, age, gender, and language and are then used as an offset in the model. Demographic data do not include information on language. To tackle this issue, the French-speaking population was calculated as the sum of the population residing in Wallonia plus 90% of the population of Brussels, and the Dutch-speaking population was calculated as the sum of Flanders residents plus 10% of the Brussels population, reflecting the Belgian language repartition. We additionally performed sensitivity analyses that excluded Brussels. The values used for the demographic offset are in eTable 1 in Supplement 1.

### Statistical Analysis

We conducted Poisson fixed-effects regressions on the count data of euthanasia cases in Belgium, examining the associations with year, age group, gender, language, reason, basis, suffering, and term of euthanasia. Since the study examines the total number of euthanasia cases over time—a count variable that has shown an increasing trend—we applied Poisson modeling, which is specifically designed for nonnegative integer outcomes and accounts for the mean-variance association inherent in such data,^37^ as applied previously on suicide count data.^38,39^

We compared 2 models. The first model estimated euthanasia occurrence without adjusting for population size, providing raw counts that identified broad trends but may have overlooked the influence of demographic patterns, leading to potential bias. The second model included an offset for population size by year, age, gender, and region, allowing calculation of euthanasia prevalence (rate relative to population at risk). This provided a more accurate depiction of how rates varied across demographic groups. Coefficients were exponentiated to obtain rate ratios (RRs) and prevalence rates (PRs).^40,41^ Since data covered all reported cases in the Belgian population, significance levels were not required (but they are provided for transparency). We replicated the model both unadjusted and fully adjusted for age, gender, and region. Poisson regressions used offsets to account for population size, but adjusting for demographic characteristics was crucial as the offset did not address within-population differences associated with outcomes.

Additional analyses included multiplicative interaction terms between years and all covariates, with marginal effects calculated to plot change over time. These are replicated using year as both a numeric and categorical variable to address the nonlinearity of time change. We also replicated analyses with 2 period subsets (2002-2015 and 2016-2023) to account for nonlinear trends. We additionally generated a baseline-adjusted model using a demographic offset fixed at baseline (year 2003) to address how demographic changes were associated with PRs. Finally, we replicated the main model marginal effects using negative binomial modeling to address potential bias in Poisson regression.

## Results

A total of 33 647 cases of euthanasia were identified. Table 1 exhibits the reported cases and percentage distribution of euthanasia by demographic characteristics, reason, basis, term of euthanasia, and type of suffering. The tableincludes all 33 647 cases, with percentages calculated from this total. Although 24 cases from before 2003 were excluded from analysis and 43 cases had missing covariate data, these cases are still reflected in the table for completeness.

**Table 1.** Reported Cases of Euthanasia by Category

Variable	No. (%) of cases (N = 33 647)
Year	
2002	24 (0.07)
2003	236 (0.70)
2004	350 (1.04)
2005	392 (1.17)
2006	432 (1.28)
2007	496 (1.47)
2008	706 (2.10)
2009	822 (2.44)
2010	956 (2.84)
2011	1134 (3.37)
2012	1430 (4.25)
2013	1815 (5.39)
2014	1933 (5.74)
2015	2023 (6.01)
2016	2028 (6.03)
2017	2315 (6.88)
2018	2362 (7.02)
2019	2658 (7.90)
2020	2446 (7.27)
2021	2700 (8.02)
2022	2966 (8.82)
2023	3423 (10.17)
Age group, y	
15-29	123 (0.37)
30-39	401 (1.19)
40-49	1145 (3.40)
50-59	3422 (10.17)
60-69	7015 (20.85)
70-79	9251 (27.49)
80-89	9063 (26.94)
≥90	3184 (9.46)
NA	43 (0.13)
Gender	
Female	16 711 (49.67)
Male	16 902 (50.23)
NA	34 (0.10)
Language	
French	7718 (22.94)
Dutch	25 895 (76.96)
NA	34 (0.10)
Reason for euthanasia	
Tumors	21 919 (65.14)
Dementia	310 (0.92)
Multimorbidity	5108 (15.18)
Nervous system diseases	2650 (7.88)
Others	375 (1.11)
Psychiatric disorders	427 (1.27)
Specific diseases	2832 (8.42)
NA	26 (0.08)
Basis for euthanasia	
Actual	33 169 (98.58)
Advanced	444 (1.32)
NA	34 (0.10)
Type of suffering	
Physical	7989 (23.74)
Both	24 627 (73.19)
Mental	995 (2.96)
NA	36 (0.11)
Term of death	
≥12 mo	4913 (14.60)
<12 mo	28 697 (85.29)
NA	37 (0.11)
Place of death	
Home	15 770 (46.87)
Hospital	12 094 (35.94)
Care home	4352 (12.93)
Palliative care	778 (2.31)
Other	617 (1.83)
NA	36 (0.11)

Abbreviation: NA, not available.

The number of euthanasia cases increased steadily, from 236 (0.70% of all reported cases) in 2003 to 1430 (4.25%) in 2012 and 3424 (10.18%) in 2023. The rise was consistent except in 2020 (during the COVID-19 pandemic), when cases decreased slightly to 2446 from 2658 the previous year. Groups aged 60 to 69 (7015 [20.85%]), 70 to 79 (9251 [27.49%]), and 80 to 89 (9063 [26.94%]) years accounted for most cases (28 513 [84.74%] 60 years or older), while those younger than 30 years constituted only 123 cases (0.37%). Gender distribution was nearly equal, with 16 711 (49.67%) females, 16 902 (50.23%) males, and 34 (0.10%) with unknown gender. Tumors were the primary cause (21 919 [65.14%]), followed by multimorbidity (5108 [15.18%]). Euthanasia due to psychiatric disorders and dementia accounted for 427 (1.27%) and 310 (0.92%) cases, respectively, affecting 737 people. Most euthanasia cases (33 169 [98.58%]) were not preplanned. Reported suffering was both physical and mental in 24 627 cases (73.19%), physical only in 7989 (23.74%), and mental only in 995 (2.96%). In 28 697 cases (85.29%), death was expected within 1 year, while in 4913 (14.60%), it was not. Most euthanasia occurred at home (15 770 [46.87%]), followed by in hospitals (12 094 [35.94%]) and care homes (4352 [12.93%]).

Our analyses focused on 33 623 valid cases. Table 2 presents the RRs, PRs, and baseline-adjusted PRs derived from the main model without interaction. The estimates indicated that the RRs and PRs were similar in the unadjusted model (both, 1.06; 95% CI, 1.06-1.06), but the PR was higher in the baseline-adjusted model (1.07; 95% CI, 1.07-1.07), suggesting that demographic composition increased the yearly rate of change by 1 percentage point. In the adjusted model, controlling for demographic characteristics, a difference of 0.02 between the RR (1.07; 95% CI, 1.07-1.07) and PR (1.05; 95% CI, 1.05-1.06) revealed that not accounting for demographics resulted in an overestimation of euthanasia cases in Belgium. The baseline-adjusted analysis confirmed that without adjusting for demographic change, the PRs and RRs remained similar. Additionally, the population 90 years or older was underrepresented in the RR (0.84; 95% CI, 0.80-0.88), but euthanasia was far more prevalent in this group when adjusting for demographics (PR, 13.19; 95% CI, 12.55-13.86). This adjustment also affected gender distribution (RR, 1.05 [95% CI, 1.02-1.07] and PR, 1.36 [95% CI, 1.33-1.39]), indicating a higher prevalence among males than among females. While previous studies reported higher euthanasia rates in the Flemish region than Wallonia, our estimates (RR, 2.45 [95% CI, 2.39-2.52]; PR, 1.51 [95% CI, 1.47-1.55]) showed that euthanasia was more prevalent in Flanders but less so than earlier studies suggested (due to an aging population in Flanders). Full results from the main model are shown in eTables 2 and 3 (model 1) in Supplement 1. Sensitivity analyses using a region and/or language variable excluding Brussels showed little difference with, for instance, a PR of 1.05 (95% CI, 1.05-1.06) for the year variable in the fully adjusted model, a difference of 0.001 units.

**Table 2.** Association of Patient Characteristics With Incidence and Prevalence of Euthanasia

Variable	Unadjusted model			Adjusted model		
	RR (95% CI)	PR (95% CI)	Baseline-offset PR (95% CI)	RR (95% CI)	PR (95% CI)	Baseline-offset PR (95% CI)
Year	1.06 (1.06-1.06)	1.06 (1.06-1.06)	1.07 (1.07-1.07)	1.07 (1.07-1.07)	1.05 (1.05-1.06)	1.07 (1.07-1.07)
Age group, y^a^						
15-29	NA	NA	NA	0.21 (0.18-0.25)	0.17 (0.14-0.20)	0.14 (0.12-0.17)
30-39	NA	NA	NA	0.29 (0.26-0.32)	0.31 (0.28-0.35)	0.25 (0.22-0.27)
40-49	NA	NA	NA	0.49 (0.46-0.52)	0.49 (0.46-0.52)	0.41 (0.39-0.44)
60-69	NA	NA	NA	1.63 (1.57-1.70)	2.00 (1.92-2.08)	2.15 (2.06-2.24)
70-79	NA	NA	NA	1.88 (1.81-1.95)	3.27 (3.14-3.40)	2.96 (2.85-3.09)
80-89	NA	NA	NA	1.80 (1.73-1.87)	5.65 (5.43-5.89)	7.29 (7.01-7.59)
≥90	NA	NA	NA	0.84 (0.80-0.88)	13.19 (12.55-13.86)	19.66 (18.71-20.66)
Gender, male^b^	NA	NA	NA	1.05 (1.02-1.07)	1.36 (1.33-1.39)	1.54 (1.51-1.57)
Language, Dutch^c^	NA	NA	NA	2.45 (2.39-2.52)	1.51 (1.47-1.55)	1.66 (1.61-1.70)
Reason for euthanasia^d^						
Dementia	0.29 (0.26-0.33)	0.31 (0.28-0.35)	0.34 (0.30-0.38)	0.20 (0.18-0.22)	0.20 (0.18-0.22)	0.20 (0.18-0.22)
Multimorbidity	0.38 (0.36-0.39)	0.49 (0.48-0.51)	0.52 (0.51-0.54)	0.31 (0.30-0.32)	0.30 (0.29-0.31)	0.30 (0.29-0.31)
Nervous system diseases	0.21 (0.20-0.22)	0.20 (0.19-0.20)	0.20 (0.19-0.21)	0.18 (0.17-0.19)	0.18 (0.17-0.19)	0.18 (0.17-0.19)
Others	0.14 (0.12-0.15)	0.13 (0.12-0.15)	0.14 (0.13-0.16)	0.10 (0.09-0.12)	0.10 (0.09-0.11)	0.10 (0.09-0.11)
Psychiatric disorders	0.37 (0.33-0.41)	0.27 (0.24-0.30)	0.26 (0.24-0.29)	0.37 (0.34-0.41)	0.39 (0.35-0.43)	0.39 (0.35-0.43)
Specific diseases	0.24 (0.23-0.25)	0.29 (0.28-0.30)	0.31 (0.30-0.32)	0.19 (0.18-0.20)	0.19 (0.18-0.20)	0.19 (0.18-0.20)
Basis for euthanasia, advanced^e^	0.23 (0.21-0.25)	0.22 (0.20-0.24)	0.22 (0.20-0.24)	0.20 (0.18-0.22)	0.20 (0.18-0.22)	0.20 (0.18-0.22)
Type of suffering^f^						
Mental and physical	1.79 (1.75-1.84)	1.79 (1.75-1.84)	1.76 (1.71-1.80)	2.01 (1.96-2.07)	2.02 (1.96-2.07)	2.01 (1.96-2.07)
Mental	0.67 (0.62-0.72)	0.70 (0.65-0.75)	0.71 (0.66-0.76)	0.63 (0.59-0.67)	0.62 (0.58-0.67)	0.63 (0.58-0.67)
Term of death, <12 mo^g^	1.80 (1.74-1.86)	1.90 (1.83-1.96)	1.90 (1.84-1.97)	1.90 (1.83-1.96)	1.91 (1.84-1.97)	1.91 (1.85-1.97)
Place of death^h^						
Hospital	0.82 (0.80-0.84)	0.85 (0.83-0.87)	0.85 (0.83-0.87)	0.82 (0.80-0.84)	0.82 (0.80-0.84)	0.82 (0.80-0.84)
Care home	0.55 (0.53-0.57)	0.80 (0.77-0.83)	0.85 (0.83-0.88)	0.46 (0.45-0.48)	0.46 (0.44-0.47)	0.46 (0.44-0.47)
Other	0.17 (0.16-0.18)	0.16 (0.15-0.18)	0.17 (0.16-0.18)	0.13 (0.12-0.14)	0.13 (0.12-0.14)	0.13 (0.12-0.14)
Palliative care	0.24 (0.23-0.26)	0.25 (0.24-0.27)	0.26 (0.24-0.28)	0.19 (0.18-0.21)	0.19 (0.18-0.21)	0.19 (0.18-0.21)
(Intercept)^i^	1.84 (1.74-1.94)	0.00 (0.00-0.00)	0.00 (0.00-0.00)	0.62 (0.58-0.66)	0.00 (0.00-0.00)	0.00 (0.00-0.00)

Abbreviations: NA, not applicable; PR, prevalence rate; RR, rate ratio.

^a^ Reference group is aged 50 to 59 years.

^b^ Reference group is female.

^c^ Reference group is French speaking (from Wallonia-Brussels).

^d^ Reference group has reason due to tumors.

^e^ Reference group is planned at actual time.

^f^ Reference group has physical suffering only.

^g^ Reference group has a term of 12 months or longer.

^h^ Reference group died at home.

^i^ The intercept represents the count without an offset, estimating rate ratios. With an offset, it represents the baseline rate and is set to 0.

The Figure shows the change in RRs and PRs over each year to capture nonlinear trends. In the unadjusted model, the RR increased steadily over time, reflecting a rise in euthanasia cases. The PR also increased but more gradually, suggesting that part of the trend was associated with population growth. When holding demographic factors constant at baseline, the PR remained lower, indicating that demographic shifts, particularly aging, were associated with the rise in euthanasia prevalence. In the adjusted model (controlling for age, gender, and region), the RR, PR, and baseline-adjusted PR overlapped for the first 15 years before diverging. This overlap suggests that early on, demographic changes had little impact on euthanasia rates, while the divergence after 2017 indicates a growing influence of factors such as aging. The shift may reflect the time needed for euthanasia regulations to be fully implemented and normalized. Around 2015, we observed the end of the regulation’s initial onset. We replicated the analysis using a linear year variable, dividing the data into 2 periods (2003-2015 and 2016-2023) (eTable 4 in Supplement 1). The RR for the onset period reached 1.12 (95% CI, 1.11-1.12), compared with 1.05 (95% CI, 1.04-1.06) in the recent period. Similarly, the PR was 1.10 (95% CI, 1.09-1.11) in the first period and 1.03 (95% CI, 1.03-1.04) in the second.

**Figure.**
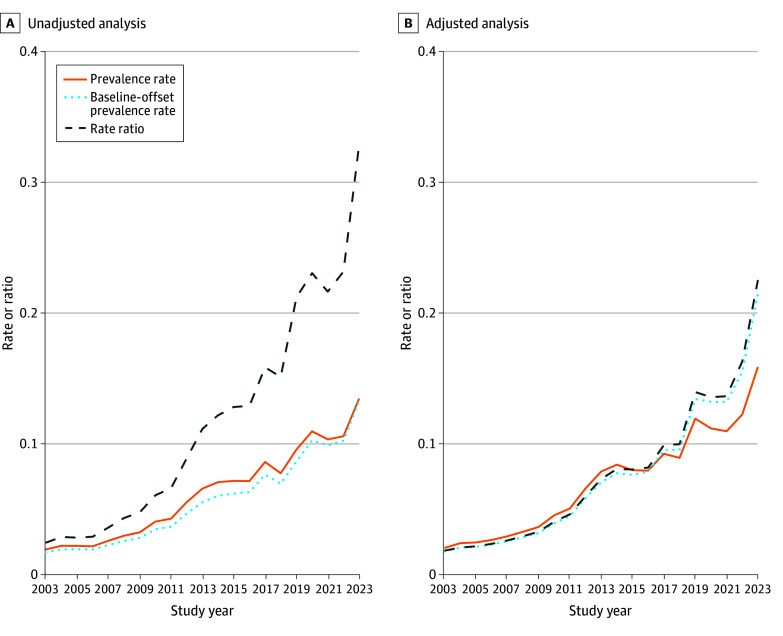
Estimated Change in Rates of Euthanasia by Year

Table 3 replicated the analyses multiple times, introducing interactions between year (as numeric) and each variable separately while adjusting for the others. Age group estimates remained stable, with constant rates for those younger than 40 years. However, PRs increased for groups aged 70 to 79 years (1.03; 95% CI, 1.03-1.04) and 80 to 89 years (1.05; 95% CI, 1.04-1.05). Among male patients, prevalence slightly declined (PR × year, 0.99; 95% CI, 0.99-1.00), though the baseline-offset analysis showed that this was largely due to demographic change (PR × year, 1.00; 95% CI, 1.00-1.00). Prevalence in the Dutch-speaking region declined (baseline-offset PR, 0.98; 95% CI, 0.98-0.99) compared with the French-speaking region (baseline-offset PR 0.99, 95% CI, 0.99-1.00). Euthanasia for multimorbidity increased relative to tumors (PR, 1.03 [95% CI, 1.02-1.04]; baseline-offset PR, 1.03 [95% CI, 1.03-1.04]), while cases of dementia, psychiatric disorders, and advanced requests remained stable. No increase was observed in cases where death was expected beyond a year (PR and baseline-offset PR, 1.01; 95% CI, 1.00-1.02). Cases with both physical and mental suffering slightly increased (PR, 1.02; 95% CI, 1.01-1.02), with a minor increase for mental suffering alone (PR, 1.00; 95% CI, 0.99-1.01). Euthanasia cases in hospitals (PR, 0.97; 95% CI, 0.96-0.97) or care homes (PR, 1.00; 95% CI, 0.99-1.01) did not increase, though a slight rise occurred in palliative care settings (PR, 1.02; 95% CI, 1.00-1.04).

**Table 3.** Interaction of Patient Characteristics With Incidence and Prevalence of Euthanasia (Fully Adjusted Model)

Variable	RR (95% CI)		PR (95% CI)			Baseline-offset PR (95% CI)	
	Main effect	Interaction × year	Main effect	Interaction × year	Main effect		Interaction × year
Age group, y^a^							
15-29	0.25 (0.15-0.42)	0.99 (0.96-1.03)	0.17 (0.10-0.29)	1.00 (0.96-1.03)	0.17 (0.10-0.27)		0.99 (0.96-1.03)
30-39	0.32 (0.25-0.41)	0.99 (0.98-1.01)	0.31 (0.24-0.40)	1.00 (0.98-1.02)	0.28 (0.21-0.36)		0.99 (0.97-1.01)
40-49	0.54 (0.46-0.64)	0.99 (0.98-1.00)	0.45 (0.38-0.53)	1.01 (0.99-1.02)	0.46 (0.39-0.54)		0.99 (0.98-1.00)
60-69	1.29 (1.16-1.44)	1.02 (1.01-1.03)	1.80 (1.62-2.01)	1.01 (1.00-1.02)	1.69 (1.52-1.88)		1.02 (1.01-1.03)
70-79	1.17 (1.05-1.29)	1.04 (1.03-1.04)	2.06 (1.85-2.29)	1.03 (1.03-1.04)	1.85 (1.66-2.05)		1.04 (1.03-1.04)
80-89	0.88 (0.79-0.99)	1.05 (1.04-1.06)	2.99 (2.68-3.34)	1.05 (1.04-1.05)	3.61 (3.24-4.03)		1.05 (1.04-1.06)
90+	0.36 (0.30-0.42)	1.06 (1.05-1.07)	10.40 (8.81-12.25)	1.02 (1.01-1.03)	7.95 (6.75-9.36)		1.06 (1.05-1.07)
Gender, male^b^	1.15 (1.08-1.23)	0.99 (0.99-1.00)	1.51 (1.41-1.61)	0.99 (0.99-1.00)	1.57 (1.47-1.67)		1.00 (1.00-1.00)
Language, Dutch^c^	2.88 (2.66-3.14)	0.99 (0.98-0.99)	1.96 (1.81-2.13)	0.98 (0.98-0.99)	1.94 (1.78-2.11)		0.99 (0.99-1.00)
Reason for euthanasia^d^							
Dementia	0.29 (0.19-0.42)	0.98 (0.95-1.00)	0.28 (0.19-0.41)	0.98 (0.96-1.00)	0.29 (0.19-0.42)		0.98 (0.95-1.00)
Multimorbidity	0.19 (0.17-0.22)	1.03 (1.02-1.04)	0.19 (0.17-0.22)	1.03 (1.02-1.04)	0.19 (0.17-0.21)		1.03 (1.03-1.04)
Nervous system diseases	0.23 (0.21-0.27)	0.98 (0.98-0.99)	0.23 (0.20-0.26)	0.98 (0.98-0.99)	0.23 (0.20-0.27)		0.98 (0.98-0.99)
Others	0.32 (0.23-0.45)	0.93 (0.91-0.95)	0.31 (0.22-0.44)	0.93 (0.91-0.95)	0.32 (0.22-0.45)		0.93 (0.91-0.95)
Psychiatric disorders	0.86 (0.62-1.19)	0.95 (0.93-0.97)	0.86 (0.62-1.18)	0.95 (0.93-0.97)	0.92 (0.66-1.27)		0.94 (0.93-0.97)
Specific diseases	0.25 (0.22-0.29)	0.98 (0.97-0.99)	0.25 (0.22-0.29)	0.98 (0.97-0.99)	0.25 (0.22-0.28)		0.98 (0.97-0.99)
Basis for euthanasia, advanced^e^	0.30 (0.23-0.38)	0.97 (0.95-0.99)	0.29 (0.23-0.37)	0.97 (0.95-0.99)	0.30 (0.24-0.38)		0.97 (0.95-0.99)
Type of suffering^f^							
Mental and physical	1.58 (1.46-1.70)	1.02 (1.01-1.02)	1.58 (1.47-1.70)	1.02 (1.01-1.02)	1.57 (1.46-1.69)		1.02 (1.01-1.02)
Mental	0.61 (0.51-0.72)	1.00 (0.99-1.01)	0.60 (0.51-0.71)	1.00 (0.99-1.01)	0.62 (0.52-0.73)		1.00 (0.99-.01)
Term of death, <12 mo^g^	1.62 (1.45-1.81)	1.01 (1.00-1.02)	1.68 (1.50-1.88)	1.01 (1.00-1.02)	1.63 (1.45-1.82)		1.01 (1.00-1.02)
Place of death^h^							
Hospital	1.31 (1.22-1.40)	0.97 (0.96-0.97)	1.30 (1.22-1.40)	0.97 (0.96-0.97)	1.30 (1.22-1.39)		0.97 (0.96-0.97)
Care home	0.46 (0.41-0.52)	1.00 (0.99-1.01)	0.46 (0.41-0.52)	1.00 (0.99-1.01)	0.44 (0.39-0.50)		1.00 (0.99-1.01)
Others	0.24 (0.18-0.30)	0.96 (0.95-0.98)	0.23 (0.18-0.30)	0.96 (0.95-0.98)	0.23 (0.18-0.29)		0.96 (0.95-0.98)
Palliative care	0.13 (0.09-0.18)	1.02 (1.00-1.04)	0.13 (0.09-0.18)	1.02 (1.00-1.04)	0.13 (0.09-0.19)		1.02 (1.00-1.04)

Abbreviations: PR, prevalence rate; RR, rate ratio.

^a^ Reference group is aged 50 to 59 years.

^b^ Reference group is female.

^c^ Reference group is French speaking (from Wallonia-Brussels).

^d^ Reference group has reason due to tumors.

^e^ Reference group is planned at actual time.

^f^ Reference group has physical suffering only.

^g^ Reference group has a term of 12 months or longer.

^h^ Reference group died at home.

All interaction effect estimates are found in eTable 2 (models 2-6) and eTable 3 (models 2-9) in Supplement 1. Marginal effects calculated for each fully adjusted models are shown in eFigures 1 and 2 in Supplement 1. Marginal effects from the negative binomial model are shown in eFigure 3 in Supplement 1, and the patterns observed during the selected period did not differ from those observed in the Poisson regression.

## Discussion

This cross-sectional study found that one-third to one-fourth of the overall increase of the prevalence of reported cases of euthanasia in Belgium during the study period could be attributed to demographic changes. While there was a steep increase in reported cases during the first 10 years following the implementation of the regulation, the rate of increase slowed down after 2015, suggesting that it took time for both the Belgian population and health care practitioners to become familiar with and adopt the practice of euthanasia. Early trends might not reflect long-term trends. We also observe a long-term shift in reported euthanasia prevalence, marked by an increase among women and a reduction in regional differences between Dutch- and French-speaking areas. Finally, the rise in cases citing multimorbidity underscores the growing complexity of health conditions among those seeking euthanasia, whereas cases associated with psychiatric disorders and dementia have remained relatively stable.

### Limitations

Although this study is, to our knowledge, the first to use administrative data on all reported cases of euthanasia in Belgium between 2002 and 2023, it has several limitations. First, the FCCEE’s data collection methods, which exclude patient identifiers, limit our ability to link euthanasia cases with socioeconomic data. Second, the absence of information on patients’ regions of residence constrains more detailed regional analysis. Third, by focusing solely on reported cases, we acknowledge that unreported instances of assisted dying exist, as evidenced in previous studies.^31,42^ Therefore, the findings from this study are limited to reported cases only. Fourth, since the dataset lacks information on the exact month of each euthanasia case, we were unable to account for a potential reduction in cases during the COVID-19 pandemic, which may have slightly biased the patterns observed from 2020 onward.

## Conclusions

While studies using the slippery slope argument often lack evidence^43,44^ or rely on descriptive statistics,^45^ this cross-sectional study demonstrates the necessity of accounting for demographic structure and changes when analyzing time-series euthanasia data. We found no evidence supporting such an argument in Belgium, where safeguards appear effective. The Belgian law has permitted euthanasia for nonterminal conditions from the start, and rates of euthanasia for psychiatric or cognitive conditions have not risen uncontrollably. We recommend that future research focus on making better use of the available data and applying greater methodological rigor, rather than relying on crude descriptions that may misrepresent actual trends.

## Notes

Supplement 1. **eTable 1.** Population Count on January 1 of Years 2002 to 2023 by Language, Sex, and Age Group **eTable 2.** Poisson Regression, not Adjusted for Demographic Characteristics **eTable 3.** Poisson Regression, Adjusted for Demographic Characteristics **eFigure 1.** Marginal Effects in the Fully Adjusted Model **eFigure 2.** Marginal Effects (Year as Categorical) **eTable 4.** Linear Trends of Year by Time Period (2003-2015 and 2016-2022), Fully Adjusted Model Without Interaction **eFigure 3.** Marginal Effects (Year as Categorical), Negative Binomial Regression

Supplement 2.
Data Sharing Statement
