# Supplementary material for: Incidence and Prevalence of Reported Euthanasia Cases in Belgium, 2002 to 2023
Source: JAMA Netw Open. 2025 Apr 23;8(4):e256841. doi: 10.1001/jamanetworkopen.2025.6841 (PMC12019508; doi:10.1001/jamanetworkopen.2025.6841)
Supplement: Supplement 1. — eTable 1. Population Count on January 1 of Years 2002 to 2023 by Language, Sex, and Age Group eTable 2. Poisson Regression, not Adjusted for Demographic Characteristics eTable 3. Poisson Regression, Adjusted for Demographic Characteristics eFigure 1. Marginal Effects in the Fully Adjusted Model eFigure 2. Marginal Effects (Year as Categorical) eTable 4. Linear Trends of Year by Time Period (2003-2015 and 2016-2022), Fully Adjusted Model Without Interaction eFigure 3. Marginal Effects (Year as Categorical), Negative Binomial Regression [file jamanetwopen-e256841-s001.pdf]

Supplementary Online Content

Wels J, Hamarat N. Incidence and prevalence of reported euthanasia cases in Belgium, 2002 to 2023. *JAMA Netw Open.* 2025;8(4):e256841. doi:10.1001/jamanetworkopen.2025.6841

eTable 1. Population Count on January 1 of Years 2002 to 2023 by Language, Sex, and Age Group
eTable 2. Poisson Regression, not Adjusted for Demographic Characteristics
eTable 3. Poisson Regression, Adjusted for Demographic Characteristics
eFigure 1. Marginal Effects in the Fully Adjusted Model
eFigure 2. Marginal Effects (Year as Categorical)
eTable 4. Linear Trends of Year by Time Period (2003-2015 and 2016-2022), Fully Adjusted Model Without Interaction
eFigure 3. Marginal Effects (Year as Categorical), Negative Binomial Regression

This supplementary material has been provided by the authors to give readers additional information about their work.

© 2025 Wels J and Hamarat N. *JAMA Network Open*.

eTable 1. Population Count on January 1 of Years 2002 to 2023 by Language, Sex, and Age Group

Language	Gender	Age group	Year	Brussels included	Brussels excluded	Year	Brussels included	Brussels excluded
FR	female	15-29	**2002**	399,434	306,945	**2014**	440,861	330,067
NL	female	15-29	**2002**	541,731	531,454	**2014**	566,178	553,868
FR	female	30-39	**2002**	312,059	241,998	**2014**	310,041	221,870
NL	female	30-39	**2002**	453,726	445,941	**2014**	411,256	401,459
FR	female	40-49	**2002**	308,436	248,853	**2014**	321,780	251,412
NL	female	40-49	**2002**	450,034	443,414	**2014**	458,862	451,043
FR	female	50-59	**2002**	257,702	207,844	**2014**	310,225	250,802
NL	female	50-59	**2002**	373,668	368,128	**2014**	462,914	456,311
FR	female	60-69	**2002**	202,791	163,746	**2014**	256,771	211,232
NL	female	60-69	**2002**	321,237	316,899	**2014**	374,373	369,313
FR	female	70-79	**2002**	207,283	166,338	**2014**	168,883	136,998
NL	female	70-79	**2002**	284,023	279,474	**2014**	281,269	277,726
FR	female	80-89	**2002**	98,347	75,437	**2014**	127,342	103,141
NL	female	80-89	**2002**	126,474	123,928	**2014**	193,749	191,060
FR	female	90+	**2002**	20,841	15,137	**2014**	28,098	21,549
NL	female	90+	**2002**	27,079	26,445	**2014**	37,774	37,046
FR	male	15-29	**2002**	407,269	317,578	**2014**	442,485	338,898
NL	male	15-29	**2002**	560,747	550,781	**2014**	579,511	568,001
FR	male	30-39	**2002**	319,100	244,472	**2014**	312,265	222,512
NL	male	30-39	**2002**	468,597	460,305	**2014**	416,679	406,706
FR	male	40-49	**2002**	307,122	248,814	**2014**	329,633	252,557
NL	male	40-49	**2002**	464,637	458,158	**2014**	471,849	463,285

© 2025 Wels J and Hamarat N. *JAMA Network Open*.

FR	male	50-59	**2002**	251,055	204,457	**2014**	301,841	244,569
NL	male	50-59	**2002**	381,891	376,713	**2014**	471,215	464,851
FR	male	60-69	**2002**	176,655	143,328	**2014**	234,217	195,161
NL	male	60-69	**2002**	300,022	296,319	**2014**	367,283	362,943
FR	male	70-79	**2002**	141,457	115,104	**2014**	128,838	105,554
NL	male	70-79	**2002**	218,525	215,597	**2014**	238,964	236,377
FR	male	80-89	**2002**	43,610	33,673	**2014**	67,883	55,260
NL	male	80-89	**2002**	66,391	65,287	**2014**	120,435	119,032
FR	male	90+	**2002**	4,681	3,384	**2014**	8,520	6,450
NL	male	90+	**2002**	7,856	7,712	**2014**	14,089	13,859
FR	female	15-29	**2003**	399,733	305,687	**2015**	441,636	330,236
NL	female	15-29	**2003**	539,673	529,223	**2015**	566,157	553,779
FR	female	30-39	**2003**	312,554	240,967	**2015**	310,929	221,787
NL	female	30-39	**2003**	447,916	439,962	**2015**	413,496	403,591
FR	female	40-49	**2003**	310,509	250,321	**2015**	319,385	248,334
NL	female	40-49	**2003**	457,094	450,406	**2015**	449,627	441,732
FR	female	50-59	**2003**	267,213	215,990	**2015**	313,237	253,008
NL	female	50-59	**2003**	384,247	378,556	**2015**	469,902	463,210
FR	female	60-69	**2003**	197,625	159,222	**2015**	262,771	216,798
NL	female	60-69	**2003**	315,590	311,323	**2015**	380,802	375,694
FR	female	70-79	**2003**	205,480	165,380	**2015**	169,204	137,160
NL	female	70-79	**2003**	287,846	283,390	**2015**	282,881	279,321
FR	female	80-89	**2003**	102,107	78,789	**2015**	126,353	102,480
NL	female	80-89	**2003**	131,180	128,589	**2015**	196,917	194,264
FR	female	90+	**2003**	21,151	15,406	**2015**	29,713	22,889
NL	female	90+	**2003**	27,805	27,167	**2015**	40,683	39,925
FR	male	15-29	**2003**	407,180	316,364	**2015**	444,104	339,691
NL	male	15-29	**2003**	558,654	548,563	**2015**	579,699	568,098
FR	male	30-39	**2003**	320,259	243,375	**2015**	311,809	221,820
NL	male	30-39	**2003**	461,647	453,104	**2015**	417,500	407,501

© 2025 Wels J and Hamarat N. *JAMA Network Open*.

FR	male	40-49	**2003**	309,497	249,813	**2015**	327,617	249,645
NL	male	40-49	**2003**	471,220	464,588	**2015**	461,680	453,016
FR	male	50-59	**2003**	260,342	212,523	**2015**	305,738	246,932
NL	male	50-59	**2003**	393,209	387,896	**2015**	478,687	472,153
FR	male	60-69	**2003**	172,827	139,966	**2015**	239,893	200,250
NL	male	60-69	**2003**	296,415	292,764	**2015**	373,706	369,301
FR	male	70-79	**2003**	141,432	115,331	**2015**	130,337	106,957
NL	male	70-79	**2003**	222,887	219,987	**2015**	242,562	239,964
FR	male	80-89	**2003**	46,305	35,967	**2015**	68,527	55,936
NL	male	80-89	**2003**	70,594	69,445	**2015**	124,424	123,025
FR	male	90+	**2003**	4,844	3,480	**2015**	9,269	7,063
NL	male	90+	**2003**	8,113	7,961	**2015**	15,473	15,228
FR	female	15-29	**2004**	401,945	306,687	**2016**	443,461	331,183
NL	female	15-29	**2004**	539,931	529,347	**2016**	567,415	554,940
FR	female	30-39	**2004**	311,257	238,822	**2016**	312,754	222,617
NL	female	30-39	**2004**	439,160	431,112	**2016**	416,499	406,484
FR	female	40-49	**2004**	313,177	252,455	**2016**	317,422	245,716
NL	female	40-49	**2004**	464,600	457,853	**2016**	441,489	433,522
FR	female	50-59	**2004**	274,492	222,523	**2016**	314,960	254,035
NL	female	50-59	**2004**	391,198	385,424	**2016**	473,796	467,027
FR	female	60-69	**2004**	196,366	157,846	**2016**	268,874	222,303
NL	female	60-69	**2004**	315,525	311,245	**2016**	388,343	383,168
FR	female	70-79	**2004**	202,075	163,107	**2016**	169,960	137,898
NL	female	70-79	**2004**	289,086	284,756	**2016**	285,038	281,476
FR	female	80-89	**2004**	105,700	81,937	**2016**	124,355	100,839
NL	female	80-89	**2004**	136,299	133,659	**2016**	198,628	196,015
FR	female	90+	**2004**	21,224	15,481	**2016**	30,787	23,916
NL	female	90+	**2004**	28,372	27,734	**2016**	42,489	41,726
FR	male	15-29	**2004**	408,377	317,123	**2016**	446,973	341,010
NL	male	15-29	**2004**	557,809	547,670	**2016**	581,899	570,125

© 2025 Wels J and Hamarat N. *JAMA Network Open*.

FR	male	30-39	**2004**	319,018	241,450	**2016**	312,678	222,454
NL	male	30-39	**2004**	451,600	442,981	**2016**	419,570	409,545
FR	male	40-49	**2004**	311,838	251,337	**2016**	325,688	246,744
NL	male	40-49	**2004**	478,382	471,660	**2016**	452,695	443,923
FR	male	50-59	**2004**	267,531	219,160	**2016**	309,193	249,005
NL	male	50-59	**2004**	400,377	395,002	**2016**	483,608	476,920
FR	male	60-69	**2004**	172,419	139,398	**2016**	245,850	205,440
NL	male	60-69	**2004**	297,828	294,159	**2016**	381,327	376,837
FR	male	70-79	**2004**	140,484	114,839	**2016**	131,714	108,286
NL	male	70-79	**2004**	225,871	223,022	**2016**	246,139	243,536
FR	male	80-89	**2004**	48,770	38,070	**2016**	68,431	55,907
NL	male	80-89	**2004**	75,009	73,820	**2016**	127,426	126,034
FR	male	90+	**2004**	5,030	3,631	**2016**	9,797	7,542
NL	male	90+	**2004**	8,233	8,078	**2016**	16,680	16,429
FR	female	15-29	**2005**	405,356	308,637	**2017**	442,457	330,812
NL	female	15-29	**2005**	542,003	531,256	**2017**	567,099	554,694
FR	female	30-39	**2005**	308,505	235,750	**2017**	314,021	224,086
NL	female	30-39	**2005**	428,817	420,733	**2017**	419,255	409,262
FR	female	40-49	**2005**	315,745	254,414	**2017**	315,964	243,696
NL	female	40-49	**2005**	471,589	464,774	**2017**	435,640	427,610
FR	female	50-59	**2005**	281,193	228,808	**2017**	315,690	254,394
NL	female	50-59	**2005**	397,976	392,155	**2017**	476,227	469,416
FR	female	60-69	**2005**	196,684	157,888	**2017**	270,055	223,238
NL	female	60-69	**2005**	317,089	312,778	**2017**	392,860	387,658
FR	female	70-79	**2005**	198,718	160,902	**2017**	175,912	143,358
NL	female	70-79	**2005**	290,885	286,683	**2017**	290,869	287,252
FR	female	80-89	**2005**	109,487	85,336	**2017**	123,105	99,933
NL	female	80-89	**2005**	142,035	139,352	**2017**	200,625	198,050
FR	female	90+	**2005**	21,880	16,102	**2017**	31,706	24,737
NL	female	90+	**2005**	29,200	28,558	**2017**	44,853	44,079

© 2025 Wels J and Hamarat N. *JAMA Network Open*.

FR	male	15-29	**2005**	410,365	318,797	**2017**	447,934	341,484
NL	male	15-29	**2005**	558,986	548,812	**2017**	583,015	571,187
FR	male	30-39	**2005**	315,616	238,166	**2017**	313,345	224,070
NL	male	30-39	**2005**	440,278	431,672	**2017**	421,843	411,924
FR	male	40-49	**2005**	314,995	253,319	**2017**	323,552	244,252
NL	male	40-49	**2005**	485,126	478,273	**2017**	445,915	437,104
FR	male	50-59	**2005**	273,561	224,879	**2017**	311,344	250,117
NL	male	50-59	**2005**	407,249	401,840	**2017**	487,053	480,250
FR	male	60-69	**2005**	173,413	140,225	**2017**	247,039	206,224
NL	male	60-69	**2005**	300,941	297,253	**2017**	385,365	380,830
FR	male	70-79	**2005**	139,333	114,074	**2017**	137,907	114,085
NL	male	70-79	**2005**	228,922	226,115	**2017**	253,603	250,956
FR	male	80-89	**2005**	51,528	40,381	**2017**	68,786	56,250
NL	male	80-89	**2005**	79,556	78,317	**2017**	130,778	129,385
FR	male	90+	**2005**	5,336	3,913	**2017**	10,159	7,895
NL	male	90+	**2005**	8,631	8,473	**2017**	17,862	17,610
FR	female	15-29	**2006**	410,716	311,863	**2018**	441,224	329,694
NL	female	15-29	**2006**	548,237	537,253	**2018**	566,867	554,475
FR	female	30-39	**2006**	306,897	233,281	**2018**	315,763	225,740
NL	female	30-39	**2006**	419,927	411,747	**2018**	421,129	411,126
FR	female	40-49	**2006**	317,585	255,622	**2018**	315,202	241,787
NL	female	40-49	**2006**	475,754	468,869	**2018**	432,982	424,825
FR	female	50-59	**2006**	288,139	235,010	**2018**	315,820	254,018
NL	female	50-59	**2006**	405,969	400,066	**2018**	476,545	469,678
FR	female	60-69	**2006**	197,600	158,814	**2018**	271,291	224,183
NL	female	60-69	**2006**	319,350	315,040	**2018**	398,444	393,210
FR	female	70-79	**2006**	194,878	157,890	**2018**	182,114	149,059
NL	female	70-79	**2006**	291,514	287,404	**2018**	295,568	291,895
FR	female	80-89	**2006**	113,872	89,319	**2018**	121,863	98,989
NL	female	80-89	**2006**	148,942	146,214	**2018**	202,879	200,337

© 2025 Wels J and Hamarat N. *JAMA Network Open*.

FR	female	90+	**2006**	21,396	15,783	**2018**	32,426	25,392
NL	female	90+	**2006**	28,829	28,205	**2018**	46,656	45,874
FR	male	15-29	**2006**	414,501	321,844	**2018**	448,148	340,604
NL	male	15-29	**2006**	564,083	553,788	**2018**	584,294	572,345
FR	male	30-39	**2006**	312,894	234,834	**2018**	314,625	225,883
NL	male	30-39	**2006**	430,497	421,824	**2018**	423,522	413,662
FR	male	40-49	**2006**	317,906	254,771	**2018**	321,980	242,270
NL	male	40-49	**2006**	489,836	482,821	**2018**	441,496	432,639
FR	male	50-59	**2006**	280,044	230,615	**2018**	312,946	250,319
NL	male	50-59	**2006**	414,942	409,450	**2018**	488,047	481,088
FR	male	60-69	**2006**	174,830	141,618	**2018**	248,286	207,124
NL	male	60-69	**2006**	304,383	300,693	**2018**	391,631	387,057
FR	male	70-79	**2006**	137,571	112,679	**2018**	144,232	119,903
NL	male	70-79	**2006**	231,376	228,610	**2018**	259,368	256,665
FR	male	80-89	**2006**	54,293	42,795	**2018**	69,248	56,789
NL	male	80-89	**2006**	84,607	83,329	**2018**	134,399	133,015
FR	male	90+	**2006**	5,238	3,840	**2018**	10,481	8,165
NL	male	90+	**2006**	8,675	8,520	**2018**	18,856	18,599
FR	female	15-29	**2007**	416,313	316,117	**2019**	440,168	327,842
NL	female	15-29	**2007**	554,122	542,989	**2019**	566,257	553,776
FR	female	30-39	**2007**	305,860	231,257	**2019**	318,666	228,318
NL	female	30-39	**2007**	413,206	404,917	**2019**	422,981	412,942
FR	female	40-49	**2007**	318,667	256,196	**2019**	314,454	239,966
NL	female	40-49	**2007**	478,243	471,302	**2019**	431,684	423,408
FR	female	50-59	**2007**	289,718	236,348	**2019**	315,468	253,110
NL	female	50-59	**2007**	410,681	404,751	**2019**	476,284	469,355
FR	female	60-69	**2007**	204,444	165,025	**2019**	273,084	225,541
NL	female	60-69	**2007**	325,536	321,156	**2019**	404,210	398,927
FR	female	70-79	**2007**	191,546	155,322	**2019**	187,787	154,348
NL	female	70-79	**2007**	292,260	288,235	**2019**	299,830	296,115

© 2025 Wels J and Hamarat N. *JAMA Network Open*.

FR	female	80-89	**2007**	118,656	93,477	**2019**	121,366	98,827
NL	female	80-89	**2007**	156,675	153,877	**2019**	205,390	202,886
FR	female	90+	**2007**	20,444	15,106	**2019**	32,932	25,871
NL	female	90+	**2007**	27,704	27,111	**2019**	48,506	47,721
FR	male	15-29	**2007**	419,216	325,446	**2019**	447,531	338,914
NL	male	15-29	**2007**	569,244	558,825	**2019**	584,879	572,810
FR	male	30-39	**2007**	311,907	232,771	**2019**	317,027	228,060
NL	male	30-39	**2007**	423,405	414,612	**2019**	424,884	414,999
FR	male	40-49	**2007**	320,102	255,519	**2019**	321,333	240,783
NL	male	40-49	**2007**	492,914	485,738	**2019**	439,423	430,473
FR	male	50-59	**2007**	281,323	231,494	**2019**	314,015	249,876
NL	male	50-59	**2007**	418,935	413,398	**2019**	487,731	480,604
FR	male	60-69	**2007**	182,315	148,453	**2019**	250,171	208,377
NL	male	60-69	**2007**	312,361	308,599	**2019**	398,263	393,619
FR	male	70-79	**2007**	136,518	111,920	**2019**	150,334	125,516
NL	male	70-79	**2007**	234,330	231,597	**2019**	264,853	262,095
FR	male	80-89	**2007**	57,152	45,231	**2019**	69,868	57,344
NL	male	80-89	**2007**	89,550	88,225	**2019**	138,028	136,636
FR	male	90+	**2007**	5,012	3,696	**2019**	10,932	8,579
NL	male	90+	**2007**	8,447	8,301	**2019**	19,968	19,707
FR	female	15-29	**2008**	421,879	319,858	**2020**	439,713	326,189
NL	female	15-29	**2008**	558,878	547,542	**2020**	567,530	554,916
FR	female	30-39	**2008**	306,324	229,712	**2020**	321,606	230,802
NL	female	30-39	**2008**	410,479	401,967	**2020**	425,263	415,174
FR	female	40-49	**2008**	319,061	255,906	**2020**	313,970	238,519
NL	female	40-49	**2008**	478,623	471,606	**2020**	431,486	423,103
FR	female	50-59	**2008**	291,209	237,543	**2020**	314,865	251,896
NL	female	50-59	**2008**	416,604	410,641	**2020**	474,420	467,423
FR	female	60-69	**2008**	211,647	171,389	**2020**	276,162	228,028
NL	female	60-69	**2008**	330,615	326,142	**2020**	411,983	406,635

© 2025 Wels J and Hamarat N. *JAMA Network Open*.

FR	female	70-79	**2008**	188,446	153,053	**2020**	192,831	159,050
NL	female	70-79	**2008**	293,953	290,020	**2020**	304,301	300,548
FR	female	80-89	**2008**	122,835	97,209	**2020**	120,747	98,583
NL	female	80-89	**2008**	164,418	161,571	**2020**	207,144	204,681
FR	female	90+	**2008**	19,478	14,412	**2020**	33,820	26,704
NL	female	90+	**2008**	26,204	25,641	**2020**	50,848	50,057
FR	male	15-29	**2008**	424,032	328,542	**2020**	447,830	337,647
NL	male	15-29	**2008**	573,963	563,353	**2020**	586,223	573,980
FR	male	30-39	**2008**	311,808	230,927	**2020**	319,310	230,039
NL	male	30-39	**2008**	419,418	410,431	**2020**	426,111	416,192
FR	male	40-49	**2008**	321,361	255,209	**2020**	320,355	239,406
NL	male	40-49	**2008**	494,062	486,712	**2020**	438,728	429,734
FR	male	50-59	**2008**	282,458	232,262	**2020**	315,007	249,389
NL	male	50-59	**2008**	425,394	419,817	**2020**	486,337	479,046
FR	male	60-69	**2008**	189,765	155,376	**2020**	253,028	210,495
NL	male	60-69	**2008**	318,374	314,553	**2020**	405,966	401,240
FR	male	70-79	**2008**	135,834	111,475	**2020**	155,544	130,417
NL	male	70-79	**2008**	238,043	235,336	**2020**	271,615	268,823
FR	male	80-89	**2008**	59,765	47,566	**2020**	70,708	58,166
NL	male	80-89	**2008**	94,821	93,466	**2020**	140,792	139,398
FR	male	90+	**2008**	4,810	3,545	**2020**	11,483	9,039
NL	male	90+	**2008**	8,004	7,863	**2020**	21,247	20,975
FR	female	15-29	**2009**	426,819	322,301	**2021**	438,480	324,553
NL	female	15-29	**2009**	563,022	551,409	**2021**	565,525	552,866
FR	female	30-39	**2009**	307,072	227,957	**2021**	323,140	232,545
NL	female	30-39	**2009**	408,960	400,169	**2021**	427,375	417,309
FR	female	40-49	**2009**	319,067	254,949	**2021**	313,992	237,636
NL	female	40-49	**2009**	477,915	470,791	**2021**	431,648	423,164
FR	female	50-59	**2009**	293,471	239,024	**2021**	314,868	251,453
NL	female	50-59	**2009**	423,137	417,087	**2021**	473,161	466,115

© 2025 Wels J and Hamarat N. *JAMA Network Open*.

FR	female	60-69	**2009**	218,138	177,147	**2021**	278,529	229,721
NL	female	60-69	**2009**	335,096	330,541	**2021**	419,238	413,815
FR	female	70-79	**2009**	186,408	151,652	**2021**	198,548	164,501
NL	female	70-79	**2009**	295,813	291,951	**2021**	311,085	307,302
FR	female	80-89	**2009**	126,616	100,589	**2021**	116,710	95,231
NL	female	80-89	**2009**	172,069	169,177	**2021**	203,502	201,115
FR	female	90+	**2009**	18,633	13,800	**2021**	33,390	26,463
NL	female	90+	**2009**	25,077	24,540	**2021**	52,103	51,333
FR	male	15-29	**2009**	428,336	330,887	**2021**	447,530	336,736
NL	male	15-29	**2009**	577,595	566,767	**2021**	586,040	573,730
FR	male	30-39	**2009**	312,528	229,681	**2021**	320,968	231,937
NL	male	30-39	**2009**	417,325	408,120	**2021**	427,800	417,908
FR	male	40-49	**2009**	322,104	254,068	**2021**	319,167	238,035
NL	male	40-49	**2009**	493,289	485,729	**2021**	437,572	428,557
FR	male	50-59	**2009**	284,380	233,278	**2021**	315,936	249,166
NL	male	50-59	**2009**	432,580	426,902	**2021**	485,646	478,227
FR	male	60-69	**2009**	196,986	161,904	**2021**	255,963	212,668
NL	male	60-69	**2009**	323,873	319,975	**2021**	413,509	408,698
FR	male	70-79	**2009**	135,626	111,385	**2021**	160,325	134,943
NL	male	70-79	**2009**	241,957	239,264	**2021**	278,924	276,104
FR	male	80-89	**2009**	61,970	49,456	**2021**	69,037	56,882
NL	male	80-89	**2009**	99,971	98,581	**2021**	139,310	137,959
FR	male	90+	**2009**	4,686	3,439	**2021**	11,361	8,983
NL	male	90+	**2009**	7,688	7,549	**2021**	22,168	21,904
FR	female	15-29	**2010**	430,201	324,278	**2022**	439,854	324,485
NL	female	15-29	**2010**	563,872	552,103	**2022**	568,373	555,554
FR	female	30-39	**2010**	308,095	226,639	**2022**	325,080	234,747
NL	female	30-39	**2010**	408,477	399,426	**2022**	431,150	421,113
FR	female	40-49	**2010**	319,308	253,821	**2022**	313,192	236,462
NL	female	40-49	**2010**	475,865	468,589	**2022**	432,406	423,880

© 2025 Wels J and Hamarat N. *JAMA Network Open*.

FR	female	50-59	**2010**	297,580	241,879	**2022**	315,144	251,229
NL	female	50-59	**2010**	431,753	425,564	**2022**	470,650	463,548
FR	female	60-69	**2010**	224,191	182,435	**2022**	281,587	232,408
NL	female	60-69	**2010**	339,707	335,067	**2022**	427,733	422,269
FR	female	70-79	**2010**	184,361	150,055	**2022**	205,856	171,148
NL	female	70-79	**2010**	296,630	292,818	**2022**	320,453	316,597
FR	female	80-89	**2010**	128,464	102,430	**2022**	113,460	92,681
NL	female	80-89	**2010**	177,702	174,809	**2022**	199,425	197,116
FR	female	90+	**2010**	19,628	14,697	**2022**	34,460	27,408
NL	female	90+	**2010**	26,067	25,519	**2022**	55,453	54,669
FR	male	15-29	**2010**	431,283	332,641	**2022**	448,855	336,779
NL	male	15-29	**2010**	577,703	566,743	**2022**	589,283	576,830
FR	male	30-39	**2010**	313,248	228,478	**2022**	322,915	234,233
NL	male	30-39	**2010**	415,962	406,543	**2022**	431,121	421,267
FR	male	40-49	**2010**	323,556	253,574	**2022**	317,103	236,392
NL	male	40-49	**2010**	491,092	483,316	**2022**	438,375	429,407
FR	male	50-59	**2010**	287,481	235,321	**2022**	317,147	249,540
NL	male	50-59	**2010**	440,164	434,368	**2022**	483,060	475,548
FR	male	60-69	**2010**	203,707	168,002	**2022**	259,644	215,427
NL	male	60-69	**2010**	330,413	326,446	**2022**	421,835	416,922
FR	male	70-79	**2010**	135,566	111,407	**2022**	166,257	140,433
NL	male	70-79	**2010**	244,309	241,625	**2022**	287,992	285,123
FR	male	80-89	**2010**	63,632	51,017	**2022**	67,702	55,856
NL	male	80-89	**2010**	104,043	102,641	**2022**	137,345	136,029
FR	male	90+	**2010**	5,173	3,847	**2022**	11,900	9,469
NL	male	90+	**2010**	8,440	8,293	**2022**	23,977	23,707
FR	female	15-29	**2011**	434,396	326,090	**2023**	444,924	325,085
NL	female	15-29	**2011**	565,728	553,694	**2023**	577,241	563,926
FR	female	30-39	**2011**	310,676	226,458	**2023**	329,543	237,569
NL	female	30-39	**2011**	409,047	399,689	**2023**	440,060	429,841

© 2025 Wels J and Hamarat N. *JAMA Network Open*.

FR	female	40-49	**2011**	320,734	253,565	**2023**	314,463	236,377
NL	female	40-49	**2011**	475,250	467,787	**2023**	437,771	429,095
FR	female	50-59	**2011**	301,434	244,232	**2023**	316,990	251,879
NL	female	50-59	**2011**	439,982	433,626	**2023**	468,445	461,210
FR	female	60-69	**2011**	232,055	189,152	**2023**	284,197	234,483
NL	female	60-69	**2011**	347,845	343,078	**2023**	436,107	430,583
FR	female	70-79	**2011**	180,312	146,640	**2023**	212,988	177,483
NL	female	70-79	**2011**	292,626	288,885	**2023**	329,775	325,830
FR	female	80-89	**2011**	129,291	103,396	**2023**	110,983	90,596
NL	female	80-89	**2011**	182,417	179,540	**2023**	197,044	194,779
FR	female	90+	**2011**	22,319	16,824	**2023**	34,657	27,678
NL	female	90+	**2011**	29,260	28,649	**2023**	57,027	56,252
FR	male	15-29	**2011**	435,536	334,535	**2023**	452,795	337,633
NL	male	15-29	**2011**	579,702	568,480	**2023**	596,278	583,482
FR	male	30-39	**2011**	315,695	228,040	**2023**	326,341	236,316
NL	male	30-39	**2011**	416,275	406,536	**2023**	437,987	427,984
FR	male	40-49	**2011**	326,251	253,615	**2023**	316,074	234,963
NL	male	40-49	**2011**	490,571	482,500	**2023**	441,787	432,775
FR	male	50-59	**2011**	291,578	237,818	**2023**	318,778	250,051
NL	male	50-59	**2011**	448,568	442,595	**2023**	479,069	471,433
FR	male	60-69	**2011**	211,423	174,831	**2023**	262,945	217,770
NL	male	60-69	**2011**	339,222	335,156	**2023**	429,841	424,822
FR	male	70-79	**2011**	134,080	110,007	**2023**	172,208	145,706
NL	male	70-79	**2011**	242,772	240,097	**2023**	297,438	294,493
FR	male	80-89	**2011**	64,896	52,306	**2023**	67,064	55,322
NL	male	80-89	**2011**	108,648	107,249	**2023**	137,541	136,236
FR	male	90+	**2011**	6,140	4,596	**2023**	12,335	9,780
NL	male	90+	**2011**	10,011	9,839	**2023**	25,057	24,773
FR	female	15-29	**2012**	436,979	327,371	2,024	448,555	325,904
NL	female	15-29	**2012**	566,942	554,763	2,024	582,847	569,219

© 2025 Wels J and Hamarat N. *JAMA Network Open*.

FR	female	30-39	**2012**	311,289	225,217	2,024	331,614	238,655
NL	female	30-39	**2012**	409,066	399,502	2,024	444,010	433,681
FR	female	40-49	**2012**	321,823	253,268	2,024	313,871	235,246
NL	female	40-49	**2012**	471,983	464,366	2,024	442,102	433,366
FR	female	50-59	**2012**	305,265	246,931	2,024	316,247	250,595
NL	female	50-59	**2012**	448,824	442,342	2,024	461,915	454,620
FR	female	60-69	**2012**	241,431	197,320	2,024	286,949	236,810
NL	female	60-69	**2012**	358,667	353,766	2,024	443,949	438,378
FR	female	70-79	**2012**	174,536	141,826	2,024	218,682	182,620
NL	female	70-79	**2012**	285,864	282,230	2,024	335,866	331,859
FR	female	80-89	**2012**	129,436	103,997	2,024	110,832	90,622
NL	female	80-89	**2012**	187,188	184,361	2,024	198,600	196,354
FR	female	90+	**2012**	24,763	18,832	2,024	34,707	27,740
NL	female	90+	**2012**	32,562	31,903	2,024	58,946	58,172
FR	male	15-29	**2012**	438,279	336,242	2,024	457,536	339,840
NL	male	15-29	**2012**	579,537	568,200	2,024	605,183	592,106
FR	male	30-39	**2012**	315,134	226,066	2,024	328,519	237,749
NL	male	30-39	**2012**	416,713	406,817	2,024	442,323	432,237
FR	male	40-49	**2012**	328,431	253,813	2,024	314,518	233,863
NL	male	40-49	**2012**	487,094	478,803	2,024	444,153	435,191
FR	male	50-59	**2012**	295,667	240,592	2,024	319,055	249,305
NL	male	50-59	**2012**	456,722	450,603	2,024	472,504	464,754
FR	male	60-69	**2012**	219,823	182,165	2,024	266,569	220,404
NL	male	60-69	**2012**	350,493	346,309	2,024	438,259	433,130
FR	male	70-79	**2012**	130,894	107,363	2,024	177,422	150,552
NL	male	70-79	**2012**	238,653	236,038	2,024	304,137	301,151
FR	male	80-89	**2012**	66,334	53,702	2,024	67,979	56,016
NL	male	80-89	**2012**	113,409	112,005	2,024	140,713	139,384
FR	male	90+	**2012**	7,246	5,416	2,024	12,611	10,084
NL	male	90+	**2012**	11,577	11,374	2,024	26,362	26,081

© 2025 Wels J and Hamarat N. *JAMA Network Open*.

FR	female	15-29	**2013**	438,947	328,484
NL	female	15-29	**2013**	566,981	554,707
FR	female	30-39	**2013**	311,298	223,667
NL	female	30-39	**2013**	409,415	399,678
FR	female	40-49	**2013**	322,958	253,181
NL	female	40-49	**2013**	466,790	459,037
FR	female	50-59	**2013**	307,468	248,587
NL	female	50-59	**2013**	455,637	449,095
FR	female	60-69	**2013**	250,050	204,949
NL	female	60-69	**2013**	368,349	363,338
FR	female	70-79	**2013**	170,053	138,000
NL	female	70-79	**2013**	281,105	277,544
FR	female	80-89	**2013**	128,999	104,163
NL	female	80-89	**2013**	191,218	188,458
FR	female	90+	**2013**	26,597	20,384
NL	female	90+	**2013**	34,963	34,273
FR	male	15-29	**2013**	440,970	337,726
NL	male	15-29	**2013**	579,677	568,205
FR	male	30-39	**2013**	314,351	224,258
NL	male	30-39	**2013**	416,501	406,491
FR	male	40-49	**2013**	330,085	253,886
NL	male	40-49	**2013**	480,832	472,365
FR	male	50-59	**2013**	298,539	242,341
NL	male	50-59	**2013**	463,576	457,332
FR	male	60-69	**2013**	227,882	189,264
NL	male	60-69	**2013**	360,541	356,250
FR	male	70-79	**2013**	128,760	105,553
NL	male	70-79	**2013**	236,978	234,399
FR	male	80-89	**2013**	67,260	54,616
NL	male	80-89	**2013**	117,269	115,864

© 2025 Wels J and Hamarat N. *JAMA Network Open*.

FR	male	90+	**2013**	7,914	5,920
NL	male	90+	**2013**	12,838	12,616

© 2025 Wels J and Hamarat N. *JAMA Network Open*.

eTable 2. Poisson Regression, not Adjusted for Demographic Characteristics

Model 1. Main model

	Incidence rate ratio			Prevalence ratio			Prevalence ratio (sensitivity check)								
				Includng Brussels			1. excluding Brussels			2. baseline values, including Brussels			3. baseline values, excluding Brussels		
	RR	2.5 %	97.5 %	PR	2.5 %	97.5 %	PR	2.5 %	97.5 %	PR	2.5 %	97.5 %	PR	2.5 %	97.5 %
(Intercept)	1.840	1.744	1.941	0.000	0.000	0.000	0.000	0.000	0.000	0.000	0.000	0.000	0.000	0.000	0.000
year	1.059	1.057	1.061	1.059	1.056	1.061	1.060	1.058	1.063	1.069	1.066	1.071	1.070	1.068	1.073
Reason: Dementia	0.289	0.257	0.325	0.313	0.278	0.351	0.301	0.267	0.338	0.338	0.300	0.380	0.327	0.290	0.367
Reason: Multimorbidity	0.375	0.363	0.387	0.491	0.475	0.507	0.479	0.464	0.495	0.522	0.505	0.539	0.514	0.498	0.531
Reason: Nervous system diseases	0.213	0.204	0.222	0.195	0.187	0.203	0.193	0.185	0.201	0.199	0.191	0.208	0.197	0.189	0.206
Reason: Others	0.135	0.122	0.149	0.134	0.121	0.149	0.129	0.117	0.143	0.141	0.127	0.156	0.136	0.122	0.150
Reason: Psychiatric disorders	0.369	0.332	0.409	0.272	0.244	0.301	0.259	0.233	0.288	0.263	0.237	0.293	0.251	0.225	0.279
Reason: Specific diseases	0.241	0.232	0.251	0.291	0.279	0.303	0.283	0.272	0.295	0.307	0.295	0.320	0.302	0.290	0.314
Basis: advanced	0.226	0.205	0.248	0.216	0.196	0.237	0.215	0.195	0.235	0.222	0.202	0.243	0.221	0.201	0.243
Suffering: both	1.792	1.746	1.839	1.793	1.747	1.840	1.823	1.776	1.871	1.757	1.713	1.804	1.784	1.738	1.830
Suffering: mental	0.666	0.620	0.715	0.702	0.653	0.753	0.705	0.655	0.757	0.708	0.659	0.761	0.713	0.663	0.766
Term: Short term	1.797	1.737	1.859	1.896	1.832	1.962	1.901	1.837	1.968	1.899	1.835	1.966	1.903	1.838	1.970
Place: Hospital	0.819	0.800	0.838	0.846	0.826	0.867	0.855	0.835	0.875	0.850	0.831	0.871	0.859	0.839	0.880
Place: Nursing home	0.551	0.533	0.570	0.797	0.771	0.825	0.791	0.765	0.818	0.853	0.825	0.883	0.855	0.826	0.884
Place: Other	0.169	0.155	0.183	0.163	0.150	0.177	0.159	0.147	0.173	0.169	0.156	0.183	0.165	0.152	0.179
Place: Palliative care	0.244	0.227	0.262	0.254	0.236	0.272	0.248	0.231	0.267	0.263	0.244	0.282	0.258	0.240	0.278

© 2025 Wels J and Hamarat N. *JAMA Network Open*.

Model 2. Interaction between year and reason for euthanasia

	Incidence rate ratio			Prevalence ratio						Prevalence ratio (sensitivity check)					
				Includng Brussels			1. excluding Brussels			2. baseline values, including Brussels			3. baseline values, excluding Brussels		
	RR	2.5 %	97.5 %	PR	2.5 %	97.5 %	PR	2.5 %	97.5 %	PR	2.5 %	97.5 %	PR	2.5 %	97.5 %
(Intercept)	1.809	1.710	1.913	0.000	0.000	0.000	0.000	0.000	0.000	0.000	0.000	0.000	0.000	0.000	0.000
year	1.060	1.058	1.063	1.060	1.058	1.063	1.062	1.059	1.065	1.070	1.067	1.072	1.071	1.069	1.074
Reason: Dementia	0.437	0.292	0.638	0.579	0.391	0.839	0.556	0.376	0.805	0.595	0.403	0.860	0.568	0.385	0.821
Reason: Multimorbidity	0.263	0.236	0.294	0.365	0.327	0.407	0.355	0.318	0.396	0.371	0.332	0.413	0.360	0.322	0.401
Reason: Nervous system diseases	0.260	0.228	0.296	0.238	0.209	0.271	0.233	0.204	0.265	0.238	0.209	0.271	0.233	0.204	0.265
Reason: Others	0.338	0.238	0.472	0.311	0.221	0.430	0.298	0.212	0.412	0.322	0.229	0.444	0.308	0.219	0.425
Reason: Psychiatric disorders	0.757	0.548	1.035	0.544	0.393	0.744	0.511	0.369	0.700	0.550	0.397	0.753	0.516	0.372	0.706
Reason: Specific diseases	0.346	0.304	0.394	0.431	0.378	0.490	0.415	0.364	0.472	0.431	0.378	0.490	0.415	0.364	0.471
Basis: advanced	0.226	0.205	0.248	0.216	0.196	0.237	0.215	0.195	0.236	0.222	0.202	0.243	0.221	0.201	0.243
Suffering: both	1.792	1.747	1.839	1.792	1.746	1.839	1.822	1.776	1.870	1.757	1.712	1.803	1.784	1.738	1.831
Suffering: mental	0.667	0.621	0.716	0.703	0.654	0.755	0.706	0.657	0.758	0.710	0.661	0.763	0.715	0.665	0.768
Term: Short term	1.801	1.741	1.863	1.897	1.833	1.964	1.903	1.839	1.970	1.902	1.838	1.969	1.906	1.842	1.974
Place: Hospital	0.819	0.799	0.838	0.847	0.827	0.867	0.856	0.836	0.876	0.851	0.831	0.872	0.861	0.840	0.881
Place: Nursing home	0.551	0.532	0.569	0.797	0.770	0.824	0.790	0.764	0.818	0.853	0.824	0.882	0.854	0.826	0.884
Place: Other	0.168	0.155	0.182	0.163	0.150	0.177	0.159	0.147	0.172	0.169	0.155	0.183	0.165	0.152	0.179
Place: Palliative care	0.243	0.225	0.261	0.252	0.234	0.271	0.247	0.229	0.265	0.261	0.243	0.281	0.257	0.239	0.276
Reason: Dementia * Year	0.974	0.952	0.998	0.962	0.940	0.985	0.962	0.940	0.985	0.965	0.943	0.988	0.966	0.944	0.989
Reason: Multimorbidity * Year	1.022	1.016	1.029	1.019	1.012	1.025	1.019	1.012	1.026	1.022	1.015	1.029	1.023	1.016	1.030
Reason: Nervous system diseases * Year	0.987	0.979	0.995	0.987	0.979	0.995	0.987	0.979	0.996	0.988	0.980	0.996	0.989	0.981	0.997
Reason: Others * Year	0.943	0.922	0.964	0.947	0.927	0.968	0.947	0.928	0.968	0.948	0.928	0.968	0.948	0.929	0.969
Reason: Psychiatric disorders * Year	0.953	0.934	0.973	0.955	0.935	0.975	0.956	0.936	0.976	0.952	0.932	0.972	0.953	0.933	0.973
Reason: Specific diseases * Year	0.976	0.968	0.985	0.974	0.966	0.982	0.975	0.967	0.983	0.978	0.970	0.986	0.979	0.971	0.987

© 2025 Wels J and Hamarat N. *JAMA Network Open*.

Model 3. Interaction between year and basis for euthanasia

	Incidence rate ratio			Prevalence ratio Includng Brussels			1. excluding Brussels			Prevalence ratio (sensitivity check) 2. baseline values, including Brussels			3. baseline values, excluding Brussels		
	RR	2.5 %	97.5 %	PR	2.5 %	97.5 %	PR	2.5 %	97.5 %	PR	2.5 %	97.5 %	PR	2.5 %	97.5 %
(Intercept)	1.831	1.735	1.931	0.000	0.000	0.000	0.000	0.000	0.000	0.000	0.000	0.000	0.000	0.000	0.000
year	1.060	1.057	1.062	1.059	1.057	1.061	1.061	1.058	1.063	1.069	1.067	1.071	1.071	1.068	1.073
Basis: advanced	0.364	0.283	0.463	0.269	0.209	0.343	0.258	0.200	0.329	0.266	0.206	0.340	0.255	0.198	0.326
Reason: Dementia	0.289	0.257	0.325	0.313	0.278	0.351	0.301	0.267	0.338	0.338	0.300	0.380	0.327	0.290	0.367
Reason: Multimorbidity	0.375	0.363	0.387	0.491	0.475	0.507	0.479	0.464	0.495	0.522	0.505	0.539	0.514	0.498	0.531
Reason: Nervous system diseases	0.213	0.204	0.222	0.195	0.187	0.203	0.193	0.185	0.201	0.199	0.191	0.208	0.197	0.189	0.206
Reason: Others	0.135	0.122	0.149	0.134	0.121	0.149	0.129	0.117	0.143	0.140	0.126	0.156	0.136	0.122	0.150
Reason: Psychiatric disorders	0.369	0.332	0.409	0.272	0.244	0.302	0.259	0.233	0.288	0.263	0.237	0.293	0.251	0.226	0.279
Reason: Specific diseases	0.241	0.232	0.251	0.291	0.279	0.303	0.283	0.272	0.295	0.308	0.295	0.320	0.302	0.290	0.314
Suffering: both	1.787	1.742	1.834	1.791	1.745	1.838	1.821	1.775	1.869	1.756	1.711	1.802	1.782	1.737	1.829
Suffering: mental	0.664	0.618	0.713	0.701	0.652	0.753	0.704	0.655	0.756	0.708	0.658	0.760	0.713	0.663	0.765
Term: Short term	1.797	1.737	1.860	1.896	1.832	1.963	1.901	1.837	1.968	1.899	1.835	1.966	1.903	1.838	1.970
Place: Hospital	0.819	0.799	0.838	0.846	0.826	0.867	0.855	0.835	0.875	0.850	0.830	0.871	0.859	0.839	0.880
Place: Nursing home	0.551	0.533	0.570	0.797	0.771	0.825	0.791	0.764	0.818	0.853	0.825	0.883	0.855	0.826	0.884
Place: Other	0.169	0.155	0.183	0.163	0.150	0.177	0.159	0.147	0.173	0.169	0.156	0.183	0.165	0.152	0.179
Place: Palliative care	0.244	0.227	0.262	0.253	0.235	0.272	0.248	0.231	0.267	0.263	0.244	0.282	0.258	0.240	0.278
Basis: advanced * Year	0.963	0.946	0.981	0.983	0.964	1.001	0.985	0.967	1.004	0.985	0.967	1.004	0.989	0.970	1.007

© 2025 Wels J and Hamarat N. *JAMA Network Open*.

Model 4. Interaction between year and type of suffering

	Incidence rate ratio			Prevalence ratio Includng Brussels			1. excluding Brussels			Prevalence ratio (sensitivity check) 2. baseline values, including Brussels			3. baseline values, excluding Brussels		
	RR	2.5 %	97.5 %	PR	2.5 %	97.5 %	PR	2.5 %	97.5 %	PR	2.5 %	97.5 %	PR	2.5 %	97.5 %
(Intercept)	2.164	2.013	2.326	0.000	0.000	0.000	0.000	0.000	0.000	0.000	0.000	0.000	0.000	0.000	0.000
year	1.047	1.043	1.051	1.051	1.046	1.055	1.052	1.048	1.057	1.061	1.057	1.065	1.063	1.058	1.067
Suffering: both	1.419	1.318	1.528	1.532	1.424	1.648	1.553	1.444	1.671	1.510	1.404	1.625	1.530	1.422	1.646
Suffering: mental	0.682	0.574	0.806	0.762	0.643	0.898	0.758	0.640	0.894	0.762	0.644	0.898	0.759	0.641	0.894
Reason: Dementia	0.293	0.260	0.329	0.318	0.282	0.358	0.306	0.271	0.343	0.344	0.305	0.386	0.332	0.294	0.373
Reason: Multimorbidity	0.375	0.363	0.387	0.491	0.475	0.507	0.479	0.464	0.495	0.522	0.505	0.539	0.514	0.498	0.531
Reason: Nervous system diseases	0.213	0.204	0.222	0.195	0.187	0.203	0.193	0.185	0.201	0.199	0.191	0.208	0.197	0.189	0.206
Reason: Others	0.134	0.121	0.149	0.134	0.121	0.148	0.129	0.116	0.143	0.140	0.126	0.155	0.135	0.122	0.150
Reason: Psychiatric disorders	0.374	0.336	0.415	0.276	0.248	0.307	0.263	0.237	0.293	0.268	0.240	0.298	0.255	0.229	0.284
Reason: Specific diseases	0.242	0.232	0.251	0.291	0.280	0.303	0.284	0.272	0.295	0.308	0.296	0.320	0.302	0.290	0.315
Basis: advanced	0.227	0.206	0.249	0.216	0.196	0.237	0.215	0.195	0.236	0.222	0.202	0.244	0.221	0.201	0.243
Term: Short term	1.802	1.742	1.865	1.900	1.836	1.967	1.905	1.841	1.972	1.903	1.839	1.970	1.907	1.842	1.974
Place: Hospital	0.819	0.799	0.838	0.846	0.826	0.866	0.855	0.834	0.875	0.850	0.830	0.871	0.859	0.839	0.880
Place: Nursing home	0.551	0.533	0.570	0.797	0.771	0.825	0.791	0.764	0.818	0.853	0.825	0.883	0.855	0.826	0.884
Place: Other	0.168	0.155	0.182	0.163	0.150	0.177	0.159	0.147	0.172	0.169	0.155	0.183	0.165	0.152	0.179
Place: Palliative care	0.243	0.225	0.261	0.252	0.235	0.271	0.247	0.230	0.266	0.262	0.243	0.281	0.257	0.239	0.277
Suffering: both * Year	1.016	1.011	1.021	1.011	1.006	1.016	1.011	1.006	1.016	1.011	1.006	1.015	1.011	1.006	1.015
Suffering: mental * Year	0.997	0.985	1.009	0.993	0.981	1.005	0.994	0.982	1.005	0.994	0.982	1.005	0.994	0.983	1.006

© 2025 Wels J and Hamarat N. *JAMA Network Open*.

Model 5. Interaction between year and expected term of death

	Incidence rate ratio			Prevalence ratio Includng Brussels			Prevalence ratio (sensitivity check) 1. excluding Brussels			2. baseline values, including Brussels			3. baseline values, excluding Brussels		
	RR	2.5 %	97.5 %	PR	2.5 %	97.5 %	PR	2.5 %	97.5 %	PR	2.5 %	97.5 %	PR	2.5 %	97.5 %
(Intercept)	2.173	1.946	2.422	0.000	0.000	0.000	0.000	0.000	0.000	0.000	0.000	0.000	0.000	0.000	0.000
year	1.048	1.041	1.055	1.044	1.037	1.050	1.046	1.039	1.052	1.054	1.048	1.061	1.057	1.050	1.063
Term: Short term	1.496	1.340	1.673	1.474	1.321	1.647	1.490	1.335	1.665	1.495	1.340	1.670	1.517	1.359	1.695
Reason: Dementia	0.291	0.258	0.326	0.316	0.281	0.355	0.304	0.270	0.341	0.341	0.303	0.383	0.330	0.293	0.371
Reason: Multimorbidity	0.375	0.363	0.387	0.491	0.475	0.507	0.479	0.464	0.495	0.522	0.506	0.539	0.514	0.498	0.531
Reason: Nervous system diseases	0.212	0.204	0.221	0.195	0.187	0.203	0.192	0.184	0.200	0.199	0.191	0.207	0.197	0.189	0.205
Reason: Others	0.135	0.122	0.149	0.134	0.121	0.149	0.129	0.116	0.143	0.141	0.127	0.156	0.136	0.122	0.150
Reason: Psychiatric disorders	0.368	0.331	0.408	0.270	0.243	0.300	0.258	0.232	0.286	0.262	0.236	0.291	0.250	0.225	0.278
Reason: Specific diseases	0.241	0.231	0.251	0.290	0.279	0.302	0.283	0.271	0.294	0.307	0.295	0.319	0.301	0.289	0.314
Basis: advanced	0.226	0.206	0.248	0.217	0.197	0.238	0.215	0.195	0.236	0.222	0.202	0.244	0.222	0.201	0.243
Suffering: both	1.792	1.746	1.839	1.792	1.747	1.840	1.823	1.776	1.870	1.757	1.712	1.803	1.783	1.738	1.830
Suffering: mental	0.666	0.620	0.715	0.701	0.652	0.753	0.704	0.655	0.756	0.708	0.658	0.760	0.712	0.662	0.765
Place: Hospital	0.818	0.799	0.838	0.845	0.826	0.866	0.854	0.834	0.875	0.850	0.830	0.870	0.859	0.839	0.880
Place: Nursing home	0.551	0.532	0.570	0.797	0.770	0.825	0.791	0.764	0.818	0.853	0.825	0.883	0.855	0.826	0.884
Place: Other	0.169	0.155	0.183	0.163	0.150	0.177	0.159	0.147	0.173	0.169	0.156	0.183	0.165	0.152	0.179
Place: Palliative care	0.244	0.226	0.262	0.253	0.235	0.272	0.248	0.230	0.266	0.262	0.243	0.282	0.258	0.239	0.277
Term: Short term * Year	1.012	1.005	1.019	1.016	1.009	1.023	1.016	1.009	1.023	1.015	1.009	1.022	1.015	1.008	1.021

© 2025 Wels J and Hamarat N. *JAMA Network Open*.

Model 6. Interaction between year and place of death

	Incidence rate ratio			Prevalence ratio Includng Brussels			Prevalence ratio (sensitivity check) 1. excluding Brussels			2. baseline values, including Brussels			3. baseline values, excluding Brussels		
	RR	2.5 %	97.5 %	PR	2.5 %	97.5 %	PR	2.5 %	97.5 %	PR	2.5 %	97.5 %	PR	2.5 %	97.5 %
(Intercept)	1.496	1.403	1.594	0.000	0.000	0.000	0.000	0.000	0.000	0.000	0.000	0.000	0.000	0.000	0.000
year	1.074	1.070	1.077	1.074	1.071	1.077	1.076	1.072	1.079	1.084	1.080	1.087	1.085	1.082	1.088
Place: Hospital	1.278	1.194	1.368	1.298	1.213	1.389	1.305	1.219	1.396	1.292	1.208	1.383	1.298	1.213	1.389
Place: Nursing home	0.594	0.525	0.671	1.082	0.957	1.221	1.080	0.956	1.219	1.102	0.975	1.243	1.098	0.972	1.240
Place: Other	0.327	0.252	0.420	0.285	0.219	0.367	0.274	0.211	0.353	0.288	0.221	0.371	0.277	0.213	0.357
Place: Palliative care	0.235	0.168	0.322	0.226	0.162	0.307	0.210	0.151	0.287	0.235	0.169	0.319	0.219	0.157	0.298
Reason: Dementia	0.291	0.258	0.326	0.315	0.279	0.353	0.302	0.268	0.339	0.340	0.301	0.381	0.328	0.291	0.369
Reason: Multimorbidity	0.375	0.364	0.387	0.490	0.475	0.506	0.479	0.463	0.494	0.522	0.505	0.539	0.513	0.497	0.530
Reason: Nervous system diseases	0.213	0.204	0.222	0.195	0.187	0.204	0.193	0.185	0.201	0.200	0.191	0.208	0.197	0.189	0.206
Reason: Others	0.136	0.122	0.150	0.135	0.122	0.150	0.130	0.117	0.144	0.142	0.128	0.157	0.137	0.123	0.152
Reason: Psychiatric disorders	0.369	0.332	0.409	0.271	0.244	0.301	0.259	0.233	0.288	0.263	0.236	0.292	0.251	0.225	0.279
Reason: Specific diseases	0.241	0.232	0.251	0.291	0.279	0.303	0.283	0.272	0.295	0.308	0.295	0.320	0.302	0.290	0.314
Basis: advanced	0.226	0.205	0.248	0.215	0.196	0.236	0.214	0.194	0.235	0.221	0.201	0.243	0.221	0.200	0.242
Suffering: both	1.792	1.746	1.839	1.793	1.747	1.840	1.823	1.777	1.871	1.757	1.712	1.803	1.784	1.738	1.831
Suffering: mental	0.660	0.614	0.708	0.697	0.648	0.749	0.700	0.651	0.752	0.705	0.655	0.757	0.709	0.659	0.762
Term: Short term	1.806	1.746	1.868	1.904	1.839	1.971	1.909	1.844	1.976	1.906	1.841	1.973	1.909	1.844	1.977
Place: Hospital * Year	0.969	0.965	0.973	0.970	0.966	0.975	0.971	0.966	0.975	0.971	0.967	0.975	0.971	0.967	0.976
Place: Nursing home * Year	0.994	0.987	1.002	0.980	0.973	0.987	0.980	0.972	0.987	0.983	0.976	0.990	0.983	0.976	0.991
Place: Other * Year	0.956	0.941	0.972	0.963	0.947	0.980	0.964	0.948	0.980	0.965	0.949	0.981	0.966	0.950	0.982
Place: Palliative care * Year	0.999	0.983	1.017	1.004	0.987	1.021	1.006	0.990	1.024	1.004	0.987	1.021	1.007	0.990	1.024

© 2025 Wels J and Hamarat N. *JAMA Network Open*.

eTable 3. Poisson Regression, Adjusted for Demographic Characteristics

Model 1. Main model

	Incidence rate ratio			Prevalence ratio						Prevalence ratio (sensitivity check)					
				Includng Brussels			1. excluding Brussels			2. baseline values, including Brussels			3. baseline values, excluding Brussels		
	RR	2.5 %	97.5 %	PR	2.5 %	97.5 %	PR	2.5 %	97.5 %	PR	2.5 %	97.5 %	PR	2.5 %	97.5 %
(Intercept)	0.619	0.579	0.661	0.000	0.000	0.000	0.000	0.000	0.000	0.000	0.000	0.000	0.000	0.000	0.000
year	1.070	1.067	1.072	1.054	1.051	1.056	1.053	1.051	1.055	1.070	1.068	1.073	1.070	1.068	1.073
Age group: 15-29	0.212	0.176	0.253	0.165	0.137	0.197	0.168	0.139	0.200	0.142	0.118	0.170	0.144	0.120	0.172
Age group: 30-39	0.292	0.263	0.323	0.312	0.281	0.346	0.320	0.288	0.355	0.247	0.223	0.274	0.251	0.226	0.278
Age group: 40-49	0.486	0.455	0.520	0.489	0.457	0.523	0.495	0.463	0.529	0.411	0.385	0.440	0.412	0.386	0.441
Age group: 60-69	1.632	1.566	1.700	1.998	1.918	2.082	1.982	1.902	2.065	2.147	2.061	2.237	2.148	2.062	2.238
Age group: 70-79	1.878	1.805	1.954	3.266	3.139	3.398	3.236	3.111	3.368	2.964	2.849	3.085	2.965	2.850	3.086
Age group: 80-89	1.798	1.728	1.871	5.653	5.432	5.885	5.626	5.406	5.857	7.294	7.008	7.593	7.399	7.109	7.703
Age group: 90+	0.837	0.797	0.879	13.186	12.547	13.856	13.254	12.613	13.928	19.659	18.706	20.660	20.220	19.240	21.250
Gender: male	1.046	1.024	1.069	1.363	1.334	1.393	1.359	1.330	1.389	1.538	1.505	1.572	1.533	1.499	1.567
Language: NL	2.451	2.389	2.515	1.512	1.474	1.551	1.246	1.214	1.278	1.656	1.614	1.699	1.332	1.298	1.366
Reason: Dementia	0.199	0.177	0.223	0.198	0.176	0.222	0.198	0.176	0.223	0.198	0.176	0.222	0.198	0.176	0.222
Reason: Multimorbidity	0.306	0.297	0.316	0.304	0.294	0.314	0.304	0.295	0.314	0.304	0.294	0.313	0.304	0.294	0.314
Reason: Nervous system diseases	0.180	0.172	0.187	0.179	0.172	0.186	0.179	0.172	0.186	0.179	0.172	0.186	0.179	0.172	0.186
Reason: Others	0.104	0.094	0.115	0.103	0.093	0.114	0.103	0.093	0.114	0.103	0.093	0.114	0.103	0.093	0.114
Reason: Psychiatric disorders	0.373	0.336	0.413	0.388	0.350	0.430	0.388	0.350	0.430	0.387	0.349	0.429	0.388	0.349	0.430
Reason: Specific diseases	0.190	0.183	0.198	0.189	0.181	0.196	0.189	0.181	0.196	0.188	0.181	0.196	0.189	0.181	0.196
Basis: advanced	0.200	0.181	0.219	0.200	0.182	0.220	0.200	0.182	0.219	0.201	0.182	0.220	0.201	0.182	0.220
Suffering: both	2.013	1.962	2.067	2.016	1.964	2.069	2.017	1.965	2.070	2.013	1.961	2.066	2.013	1.961	2.066
Suffering: mental	0.629	0.586	0.674	0.623	0.580	0.668	0.623	0.581	0.668	0.625	0.583	0.670	0.625	0.583	0.670
Term: Short term	1.897	1.834	1.962	1.905	1.842	1.971	1.906	1.843	1.972	1.909	1.846	1.974	1.909	1.846	1.975

© 2025 Wels J and Hamarat N. *JAMA Network Open*.

Place: Hospital	0.823	0.803	0.843	0.822	0.802	0.841	0.822	0.803	0.842	0.824	0.804	0.844	0.824	0.804	0.843
Place: Nursing home	0.461	0.446	0.477	0.457	0.442	0.473	0.457	0.442	0.473	0.458	0.442	0.474	0.458	0.442	0.474
Place: Other	0.132	0.122	0.143	0.130	0.120	0.141	0.130	0.120	0.141	0.130	0.120	0.141	0.130	0.120	0.141
Place: Palliative care	0.192	0.178	0.206	0.190	0.177	0.205	0.190	0.177	0.204	0.191	0.177	0.205	0.190	0.177	0.205

© 2025 Wels J and Hamarat N. *JAMA Network Open*.

Model 2. Interaction between year and reason for euthanasia

	Incidence rate ratio			Prevalence ratio						Prevalence ratio (sensitivity check)					
				Includng Brussels			1. excluding Brussels			2. baseline values, including Brussels			3. baseline values, excluding Brussels		
	RR	2.5 %	97.5 %	PR	2.5 %	97.5 %	PR	2.5 %	97.5 %	PR	2.5 %	97.5 %	PR	2.5 %	97.5 %
(Intercept)	0.612	0.571	0.655	0.000	0.000	0.000	0.000	0.000	0.000	0.000	0.000	0.000	0.000	0.000	0.000
year	1.070	1.067	1.073	1.054	1.051	1.056	1.054	1.051	1.056	1.070	1.068	1.073	1.070	1.068	1.073
Reason: Dementia	0.285	0.191	0.418	0.282	0.188	0.412	0.282	0.188	0.413	0.290	0.194	0.424	0.290	0.194	0.424
Reason: Multimorbidity	0.193	0.172	0.215	0.194	0.174	0.217	0.195	0.175	0.218	0.186	0.167	0.208	0.186	0.167	0.208
Reason: Nervous system diseases	0.233	0.205	0.266	0.228	0.199	0.259	0.228	0.199	0.259	0.233	0.204	0.265	0.233	0.205	0.266
Reason: Others	0.321	0.225	0.450	0.312	0.219	0.439	0.312	0.218	0.438	0.318	0.222	0.447	0.318	0.223	0.447
Reason: Psychiatric disorders	0.863	0.622	1.185	0.857	0.617	1.178	0.851	0.613	1.170	0.921	0.662	1.269	0.922	0.663	1.270
Reason: Specific diseases	0.254	0.223	0.289	0.253	0.222	0.288	0.254	0.223	0.289	0.246	0.216	0.280	0.246	0.216	0.280
LanguageNL	2.453	2.391	2.517	1.513	1.474	1.552	1.246	1.215	1.279	1.658	1.616	1.701	1.333	1.299	1.368
Age group: 15-29	0.212	0.176	0.253	0.165	0.137	0.197	0.167	0.139	0.199	0.142	0.118	0.169	0.144	0.120	0.172
Age group: 30-39	0.293	0.264	0.324	0.313	0.282	0.347	0.321	0.289	0.356	0.248	0.223	0.275	0.252	0.227	0.279
Age group: 40-49	0.485	0.453	0.518	0.487	0.455	0.520	0.493	0.461	0.527	0.410	0.383	0.438	0.411	0.384	0.439
Age group: 60-69	1.640	1.574	1.709	2.009	1.928	2.093	1.992	1.912	2.076	2.158	2.071	2.248	2.159	2.072	2.250
Age group: 70-79	1.886	1.813	1.963	3.280	3.153	3.413	3.250	3.124	3.382	2.977	2.861	3.097	2.977	2.862	3.098
Age group: 80-89	1.804	1.734	1.878	5.673	5.451	5.906	5.646	5.425	5.878	7.319	7.032	7.620	7.425	7.134	7.730
Age group: 90+	0.837	0.797	0.879	13.167	12.529	13.836	13.235	12.594	13.909	19.666	18.712	20.667	20.227	19.246	21.258
Gender: male	1.046	1.024	1.069	1.364	1.335	1.394	1.360	1.331	1.390	1.539	1.506	1.573	1.534	1.500	1.568
Basis: advanced	0.200	0.181	0.219	0.200	0.182	0.219	0.200	0.181	0.219	0.200	0.182	0.220	0.201	0.182	0.220
Suffering: both	2.016	1.964	2.069	2.018	1.966	2.071	2.019	1.967	2.072	2.015	1.964	2.068	2.016	1.964	2.069
Suffering: mental	0.627	0.584	0.672	0.621	0.579	0.666	0.622	0.579	0.667	0.624	0.581	0.669	0.624	0.581	0.669
Term: Short term	1.904	1.841	1.969	1.912	1.849	1.978	1.913	1.850	1.979	1.916	1.853	1.982	1.917	1.853	1.983
Place: Hospital	0.822	0.803	0.842	0.821	0.802	0.841	0.822	0.802	0.841	0.823	0.804	0.843	0.823	0.804	0.843
Place: Nursing home	0.461	0.445	0.476	0.457	0.441	0.472	0.457	0.441	0.473	0.457	0.442	0.473	0.457	0.442	0.473

© 2025 Wels J and Hamarat N. *JAMA Network Open*.

Place: Other	0.132	0.121	0.143	0.130	0.120	0.141	0.130	0.120	0.141	0.130	0.120	0.140	0.130	0.120	0.141
Place: Palliative care	0.191	0.177	0.205	0.189	0.176	0.203	0.189	0.176	0.203	0.189	0.176	0.203	0.189	0.176	0.203
Reason: Dementia * Year	0.977	0.954	1.002	0.978	0.955	1.002	0.978	0.955	1.002	0.976	0.954	1.000	0.976	0.954	1.000
Reason: Multimorbidity * Year	1.030	1.023	1.037	1.029	1.022	1.036	1.029	1.022	1.035	1.032	1.025	1.038	1.032	1.025	1.038
Reason: Nervous system diseases * Year	0.983	0.975	0.991	0.984	0.976	0.992	0.984	0.976	0.992	0.983	0.975	0.991	0.983	0.975	0.991
Reason: Others * Year	0.931	0.910	0.952	0.932	0.911	0.953	0.932	0.911	0.953	0.930	0.910	0.952	0.930	0.910	0.952
Reason: Psychiatric disorders * Year	0.946	0.926	0.966	0.949	0.929	0.969	0.949	0.930	0.970	0.944	0.925	0.965	0.944	0.925	0.965
Reason: Specific diseases * Year	0.981	0.973	0.989	0.981	0.973	0.989	0.981	0.973	0.989	0.982	0.974	0.991	0.982	0.974	0.991

© 2025 Wels J and Hamarat N. *JAMA Network Open*.

Model 3. Interaction between year and basis for euthanasia

	Incidence rate ratio			Prevalence ratio<br>Includng Brussels			1. excluding Brussels			Prevalence ratio (sensitivity check)<br>2. baseline values, including Brussels			3. baseline values, excluding Brussels		
	RR	2.5 %	97.5 %	PR	2.5 %	97.5 %	PR	2.5 %	97.5 %	PR	2.5 %	97.5 %	PR	2.5 %	97.5 %
(Intercept)	0.616	0.576	0.658	0.000	0.000	0.000	0.000	0.000	0.000	0.000	0.000	0.000	0.000	0.000	0.000
year	1.070	1.068	1.072	1.054	1.052	1.056	1.054	1.051	1.056	1.071	1.069	1.073	1.071	1.069	1.073
Basis: advanced	0.296	0.231	0.376	0.288	0.225	0.365	0.287	0.225	0.365	0.303	0.237	0.384	0.304	0.237	0.386
Reason: Dementia	0.199	0.177	0.223	0.198	0.176	0.222	0.198	0.176	0.222	0.198	0.176	0.222	0.198	0.176	0.222
Reason: Multimorbidity	0.306	0.297	0.316	0.304	0.294	0.314	0.304	0.294	0.314	0.303	0.294	0.313	0.304	0.294	0.314
Reason: Nervous system diseases	0.180	0.172	0.187	0.179	0.172	0.186	0.179	0.172	0.186	0.179	0.172	0.186	0.179	0.172	0.186
Reason: Others	0.104	0.094	0.115	0.103	0.093	0.114	0.103	0.093	0.114	0.103	0.093	0.114	0.103	0.093	0.114
Reason: Psychiatric disorders	0.373	0.336	0.413	0.389	0.350	0.430	0.388	0.350	0.430	0.387	0.349	0.429	0.388	0.350	0.430
Reason: Specific diseases	0.190	0.183	0.198	0.189	0.181	0.196	0.189	0.181	0.197	0.188	0.181	0.196	0.189	0.181	0.196
LanguageNL	2.450	2.388	2.513	1.511	1.473	1.550	1.245	1.213	1.277	1.655	1.613	1.698	1.331	1.297	1.365
Age group: 15-29	0.212	0.176	0.253	0.165	0.138	0.197	0.168	0.139	0.200	0.142	0.118	0.170	0.144	0.120	0.172
Age group: 30-39	0.292	0.263	0.324	0.313	0.281	0.346	0.320	0.288	0.355	0.248	0.223	0.274	0.251	0.226	0.278
Age group: 40-49	0.487	0.455	0.520	0.489	0.457	0.523	0.495	0.463	0.530	0.412	0.385	0.440	0.413	0.386	0.441
Age group: 60-69	1.633	1.567	1.701	2.000	1.920	2.084	1.983	1.904	2.067	2.149	2.063	2.240	2.150	2.064	2.241
Age group: 70-79	1.879	1.806	1.955	3.267	3.141	3.400	3.237	3.112	3.369	2.966	2.851	3.086	2.967	2.852	3.087
Age group: 80-89	1.801	1.730	1.874	5.661	5.440	5.893	5.634	5.414	5.865	7.307	7.020	7.607	7.412	7.121	7.717
Age group: 90+	0.837	0.797	0.880	13.194	12.555	13.865	13.263	12.621	13.937	19.681	18.726	20.683	20.242	19.261	21.273
Gender: male	1.046	1.024	1.069	1.364	1.334	1.394	1.359	1.330	1.389	1.539	1.505	1.573	1.533	1.500	1.567
Suffering: both	2.009	1.957	2.062	2.011	1.960	2.064	2.013	1.961	2.066	2.008	1.957	2.061	2.008	1.957	2.061
Suffering: mental	0.627	0.585	0.673	0.622	0.579	0.666	0.622	0.580	0.667	0.624	0.581	0.669	0.624	0.581	0.669
Term: Short term	1.897	1.834	1.962	1.905	1.842	1.971	1.906	1.843	1.972	1.909	1.846	1.975	1.909	1.846	1.975
Place: Hospital	0.823	0.803	0.842	0.822	0.802	0.841	0.822	0.802	0.842	0.824	0.804	0.843	0.823	0.804	0.843
Place: Nursing home	0.461	0.446	0.477	0.457	0.442	0.473	0.457	0.442	0.473	0.458	0.442	0.473	0.458	0.442	0.473
Place: Other	0.132	0.122	0.143	0.130	0.120	0.141	0.130	0.120	0.141	0.130	0.120	0.141	0.130	0.120	0.141

© 2025 Wels J and Hamarat N. *JAMA Network Open*.

Place: Palliative care	0.192	0.178	0.206	0.190	0.177	0.204	0.190	0.177	0.204	0.190	0.177	0.205	0.190	0.177	0.204
Basis: advanced * Year	0.969	0.952	0.987	0.972	0.954	0.989	0.972	0.954	0.990	0.968	0.950	0.986	0.968	0.950	0.986

© 2025 Wels J and Hamarat N. *JAMA Network Open*.

Model 4. Interaction between year and type of suffering

	Incidence rate ratio			Prevalence ratio<br>Includng Brussels			1. excluding Brussels			Prevalence ratio (sensitivity check)<br>2. baseline values, including Brussels			3. baseline values, excluding Brussels		
	RR	2.5 %	97.5 %	PR	2.5 %	97.5 %	PR	2.5 %	97.5 %	PR	2.5 %	97.5 %	PR	2.5 %	97.5 %
(Intercept)	0.735	0.677	0.798	0.000	0.000	0.000	0.000	0.000	0.000	0.000	0.000	0.000	0.000	0.000	0.000
year	1.057	1.052	1.061	1.041	1.036	1.045	1.040	1.036	1.045	1.057	1.053	1.062	1.057	1.053	1.061
Suffering: both	1.575	1.464	1.696	1.577	1.465	1.697	1.578	1.467	1.699	1.566	1.455	1.686	1.565	1.454	1.685
Suffering: mental	0.606	0.510	0.717	0.604	0.508	0.714	0.604	0.508	0.714	0.615	0.517	0.727	0.615	0.518	0.728
Reason: Dementia	0.200	0.178	0.225	0.200	0.177	0.224	0.200	0.177	0.224	0.200	0.177	0.224	0.200	0.178	0.224
Reason: Multimorbidity	0.307	0.297	0.316	0.304	0.295	0.314	0.304	0.295	0.314	0.304	0.294	0.314	0.304	0.294	0.314
Reason: Nervous system diseases	0.180	0.173	0.188	0.179	0.172	0.187	0.179	0.172	0.187	0.179	0.172	0.187	0.179	0.172	0.187
Reason: Others	0.104	0.093	0.115	0.103	0.093	0.114	0.103	0.093	0.114	0.103	0.092	0.113	0.103	0.092	0.114
Reason: Psychiatric disorders	0.376	0.338	0.416	0.391	0.352	0.433	0.391	0.352	0.433	0.390	0.351	0.433	0.391	0.352	0.433
Reason: Specific diseases	0.191	0.183	0.198	0.189	0.182	0.197	0.189	0.182	0.197	0.189	0.181	0.196	0.189	0.181	0.197
LanguageNL	2.450	2.388	2.514	1.511	1.473	1.551	1.245	1.213	1.277	1.656	1.614	1.699	1.331	1.297	1.366
Age group: 15-29	0.213	0.177	0.253	0.166	0.138	0.198	0.168	0.140	0.200	0.143	0.119	0.170	0.145	0.120	0.172
Age group: 30-39	0.292	0.263	0.323	0.312	0.281	0.346	0.320	0.288	0.354	0.247	0.222	0.274	0.251	0.226	0.278
Age group: 40-49	0.487	0.455	0.521	0.490	0.458	0.524	0.496	0.463	0.530	0.412	0.385	0.440	0.413	0.386	0.442
Age group: 60-69	1.631	1.566	1.700	1.998	1.918	2.082	1.981	1.901	2.064	2.146	2.060	2.236	2.148	2.061	2.238
Age group: 70-79	1.881	1.808	1.957	3.270	3.143	3.403	3.240	3.115	3.372	2.969	2.854	3.089	2.970	2.854	3.090
Age group: 80-89	1.802	1.731	1.875	5.666	5.444	5.898	5.639	5.418	5.870	7.310	7.023	7.610	7.415	7.124	7.720
Age group: 90+	0.838	0.798	0.881	13.202	12.563	13.873	13.270	12.628	13.945	19.708	18.752	20.711	20.271	19.288	21.304
Gender: male	1.046	1.024	1.068	1.363	1.334	1.393	1.359	1.330	1.389	1.538	1.505	1.573	1.533	1.499	1.567
Basis: advanced	0.201	0.182	0.220	0.201	0.182	0.221	0.201	0.182	0.220	0.201	0.183	0.221	0.202	0.183	0.221
Term: Short term	1.902	1.839	1.968	1.910	1.847	1.976	1.911	1.848	1.977	1.914	1.851	1.980	1.915	1.851	1.980
Place: Hospital	0.822	0.803	0.842	0.821	0.802	0.841	0.821	0.802	0.841	0.823	0.804	0.843	0.823	0.804	0.843
Place: Nursing home	0.461	0.446	0.477	0.457	0.442	0.473	0.458	0.442	0.473	0.458	0.442	0.474	0.458	0.442	0.474
Place: Other	0.132	0.122	0.143	0.130	0.120	0.141	0.130	0.120	0.141	0.130	0.120	0.141	0.130	0.120	0.141

© 2025 Wels J and Hamarat N. *JAMA Network Open*.

Place: Palliative care	0.191	0.178	0.205	0.190	0.176	0.204	0.190	0.176	0.204	0.190	0.176	0.204	0.190	0.176	0.204
Suffering: both * Year	1.017	1.012	1.022	1.017	1.012	1.022	1.017	1.012	1.022	1.017	1.013	1.022	1.018	1.013	1.022
Suffering: mental * Year	1.002	0.990	1.014	1.001	0.989	1.013	1.001	0.989	1.013	1.000	0.988	1.012	1.000	0.988	1.012

© 2025 Wels J and Hamarat N. *JAMA Network Open*.

Model 5. Interaction between year and expected term of death

				Prevalence ratio						Prevalence ratio (sensitivity check)					
	Incidence rate ratio			Includng Brussels			1. excluding Brussels			2. baseline values, including Brussels			3. baseline values, excluding Brussels		
	RR	2.5 %	97.5 %	PR	2.5 %	97.5 %	PR	2.5 %	97.5 %	PR	2.5 %	97.5 %	PR	2.5 %	97.5 %
(Intercept)	0.715	0.635	0.804	0.000	0.000	0.000	0.000	0.000	0.000	0.000	0.000	0.000	0.000	0.000	0.000
year	1.060	1.053	1.067	1.046	1.039	1.053	1.045	1.038	1.052	1.061	1.054	1.068	1.061	1.054	1.068
Term: Short term	1.620	1.448	1.814	1.675	1.497	1.876	1.674	1.497	1.875	1.627	1.454	1.822	1.629	1.456	1.824
Reason: Dementia	0.200	0.177	0.224	0.199	0.177	0.223	0.199	0.177	0.223	0.199	0.176	0.223	0.199	0.177	0.223
Reason: Multimorbidity	0.306	0.297	0.316	0.304	0.294	0.314	0.304	0.295	0.314	0.304	0.294	0.313	0.304	0.294	0.314
Reason: Nervous system diseases	0.180	0.172	0.187	0.179	0.171	0.186	0.179	0.171	0.186	0.179	0.171	0.186	0.179	0.171	0.186
Reason: Others	0.104	0.094	0.115	0.103	0.093	0.114	0.103	0.093	0.114	0.103	0.093	0.114	0.103	0.093	0.114
Reason: Psychiatric disorders	0.372	0.335	0.412	0.388	0.349	0.430	0.388	0.349	0.429	0.387	0.348	0.428	0.387	0.349	0.429
Reason: Specific diseases	0.190	0.183	0.198	0.189	0.181	0.196	0.189	0.181	0.196	0.188	0.181	0.196	0.188	0.181	0.196
LanguageNL	2.450	2.388	2.514	1.511	1.473	1.550	1.245	1.213	1.277	1.655	1.613	1.698	1.331	1.297	1.366
Age group: 15-29	0.212	0.176	0.252	0.165	0.137	0.197	0.167	0.139	0.199	0.142	0.118	0.169	0.144	0.120	0.172
Age group: 30-39	0.291	0.262	0.323	0.312	0.281	0.346	0.320	0.288	0.354	0.247	0.222	0.273	0.251	0.226	0.278
Age group: 40-49	0.486	0.454	0.519	0.489	0.457	0.523	0.495	0.463	0.529	0.411	0.384	0.439	0.412	0.385	0.440
Age group: 60-69	1.632	1.566	1.700	1.999	1.918	2.083	1.982	1.902	2.065	2.147	2.061	2.237	2.148	2.062	2.239
Age group: 70-79	1.876	1.803	1.952	3.264	3.137	3.396	3.234	3.109	3.365	2.961	2.847	3.082	2.962	2.848	3.083
Age group: 80-89	1.796	1.726	1.870	5.648	5.427	5.880	5.621	5.401	5.852	7.287	7.001	7.587	7.392	7.102	7.696
Age group: 90+	0.836	0.796	0.878	13.170	12.532	13.839	13.238	12.597	13.911	19.628	18.676	20.628	20.188	19.209	21.216
Gender: male	1.046	1.024	1.069	1.364	1.334	1.394	1.359	1.330	1.389	1.539	1.505	1.573	1.533	1.500	1.567
Basis: advanced	0.200	0.182	0.219	0.200	0.182	0.220	0.200	0.182	0.220	0.201	0.182	0.220	0.201	0.183	0.221
Suffering: both	2.013	1.962	2.066	2.015	1.964	2.068	2.016	1.965	2.070	2.012	1.961	2.065	2.013	1.961	2.066
Suffering: mental	0.629	0.586	0.674	0.623	0.580	0.668	0.623	0.581	0.668	0.625	0.583	0.670	0.625	0.583	0.670
Place: Hospital	0.822	0.803	0.842	0.821	0.802	0.841	0.822	0.802	0.841	0.823	0.804	0.843	0.823	0.804	0.843
Place: Nursing home	0.461	0.446	0.477	0.457	0.442	0.473	0.457	0.442	0.473	0.458	0.442	0.473	0.458	0.442	0.473

© 2025 Wels J and Hamarat N. *JAMA Network Open*.

Place: Other	0.132	0.122	0.143	0.130	0.120	0.141	0.130	0.120	0.141	0.130	0.120	0.141	0.130	0.120	0.141
Place: Palliative care	0.192	0.178	0.206	0.190	0.177	0.204	0.190	0.177	0.204	0.190	0.177	0.205	0.190	0.177	0.205
Term: Short term * Year	1.010	1.003	1.017	1.008	1.001	1.015	1.008	1.001	1.015	1.010	1.003	1.017	1.010	1.003	1.017

© 2025 Wels J and Hamarat N. *JAMA Network Open*.

Model 6. Interaction between year and place of death

	Incidence rate ratio			Prevalence ratio<br>Includng Brussels			1. excluding Brussels			Prevalence ratio (sensitivity check)<br>2. baseline values, including Brussels			3. baseline values, excluding Brussels		
	RR	2.5 %	97.5 %	PR	2.5 %	97.5 %	PR	2.5 %	97.5 %	PR	2.5 %	97.5 %	PR	2.5 %	97.5 %
(Intercept)	0.504	0.468	0.543	0.000	0.000	0.000	0.000	0.000	0.000	0.000	0.000	0.000	0.000	0.000	0.000
year	1.084	1.081	1.088	1.068	1.065	1.071	1.068	1.064	1.071	1.085	1.081	1.088	1.085	1.081	1.088
Place: Hospital	1.309	1.223	1.401	1.303	1.218	1.395	1.304	1.218	1.395	1.302	1.217	1.393	1.302	1.216	1.393
Place: Nursing home	0.459	0.406	0.518	0.464	0.410	0.524	0.466	0.412	0.526	0.441	0.390	0.498	0.440	0.390	0.497
Place: Other	0.236	0.183	0.302	0.230	0.178	0.295	0.230	0.177	0.295	0.228	0.176	0.292	0.228	0.176	0.292
Place: Palliative care	0.133	0.093	0.184	0.130	0.091	0.180	0.130	0.091	0.180	0.133	0.093	0.185	0.133	0.094	0.185
Reason: Dementia	0.200	0.178	0.224	0.199	0.177	0.224	0.200	0.177	0.224	0.199	0.177	0.223	0.199	0.177	0.224
Reason: Multimorbidity	0.306	0.297	0.316	0.304	0.294	0.314	0.304	0.294	0.314	0.304	0.294	0.313	0.304	0.294	0.314
Reason: Nervous system diseases	0.180	0.172	0.187	0.179	0.172	0.186	0.179	0.172	0.186	0.179	0.172	0.186	0.179	0.172	0.186
Reason: Others	0.105	0.094	0.116	0.104	0.093	0.115	0.104	0.094	0.115	0.103	0.093	0.115	0.104	0.093	0.115
Reason: Psychiatric disorders	0.372	0.336	0.412	0.388	0.349	0.430	0.388	0.349	0.429	0.387	0.348	0.428	0.388	0.349	0.429
Reason: Specific diseases	0.190	0.183	0.198	0.189	0.181	0.196	0.189	0.181	0.196	0.189	0.181	0.196	0.189	0.181	0.196
LanguageNL	2.456	2.394	2.520	1.515	1.476	1.554	1.248	1.216	1.281	1.659	1.617	1.703	1.334	1.300	1.369
Agegroup15-29	0.212	0.176	0.253	0.165	0.137	0.197	0.168	0.139	0.200	0.142	0.118	0.169	0.144	0.120	0.171
Agegroup30-39	0.290	0.261	0.321	0.310	0.279	0.344	0.318	0.286	0.352	0.246	0.221	0.272	0.250	0.225	0.276
Agegroup40-49	0.485	0.453	0.519	0.487	0.455	0.521	0.493	0.461	0.527	0.410	0.383	0.438	0.411	0.384	0.439
Agegroup60-69	1.628	1.563	1.696	1.995	1.915	2.079	1.978	1.899	2.061	2.143	2.057	2.233	2.144	2.058	2.234
Agegroup70-79	1.880	1.808	1.957	3.269	3.143	3.402	3.240	3.114	3.371	2.969	2.854	3.090	2.970	2.855	3.091
Agegroup80-89	1.802	1.731	1.875	5.667	5.445	5.900	5.640	5.419	5.872	7.313	7.026	7.613	7.418	7.127	7.723
Agegroup90+	0.837	0.797	0.879	13.184	12.546	13.854	13.253	12.611	13.927	19.680	18.726	20.682	20.241	19.259	21.271
Gendermale	1.045	1.023	1.068	1.362	1.333	1.392	1.358	1.329	1.388	1.537	1.504	1.571	1.531	1.498	1.565
basisadvanced	0.199	0.181	0.219	0.200	0.181	0.219	0.199	0.181	0.219	0.200	0.182	0.220	0.200	0.182	0.220
sufferingboth	2.016	1.964	2.069	2.018	1.966	2.071	2.019	1.968	2.072	2.015	1.963	2.068	2.015	1.964	2.069
sufferingmental	0.621	0.578	0.665	0.615	0.573	0.659	0.615	0.573	0.660	0.617	0.575	0.661	0.617	0.575	0.661

© 2025 Wels J and Hamarat N. *JAMA Network Open*.

termShort term	1.905	1.842	1.970	1.913	1.849	1.978	1.914	1.850	1.979	1.916	1.852	1.982	1.916	1.853	1.982
year:PlaceHospital	0.968	0.963	0.972	0.968	0.964	0.972	0.968	0.964	0.972	0.968	0.964	0.973	0.968	0.964	0.973
year:PlaceNursing home	0.999	0.992	1.007	0.998	0.991	1.006	0.998	0.990	1.005	1.002	0.994	1.009	1.002	0.994	1.009
year:PlaceOther	0.962	0.946	0.978	0.962	0.947	0.978	0.962	0.947	0.978	0.963	0.947	0.979	0.963	0.947	0.979
year:PlacePalliative care	1.018	1.000	1.037	1.019	1.001	1.038	1.019	1.001	1.038	1.018	1.000	1.037	1.017	1.000	1.037

© 2025 Wels J and Hamarat N. *JAMA Network Open*.

Model 7. Interaction between year and age group

	Incidence rate ratio			Prevalence ratio Includng Brussels			Prevalence ratio (sensitivity check) 1. excluding Brussels			2. baseline values, including Brussels			3. baseline values, excluding Brussels		
	RR	2.5 %	97.5 %	PR	2.5 %	97.5 %	PR	2.5 %	97.5 %	PR	2.5 %	97.5 %	PR	2.5 %	97.5 %
(Intercept)	0.929	0.841	1.025	0.000	0.000	0.000	0.000	0.000	0.000	0.000	0.000	0.000	0.000	0.000	0.000
year	1.038	1.032	1.044	1.028	1.022	1.035	1.029	1.023	1.035	1.039	1.033	1.045	1.039	1.033	1.045
Age group: 15-29	0.253	0.148	0.417	0.174	0.102	0.287	0.177	0.103	0.291	0.167	0.097	0.274	0.169	0.098	0.278
Age group: 30-39	0.319	0.245	0.412	0.310	0.238	0.399	0.316	0.243	0.408	0.275	0.211	0.355	0.279	0.214	0.360
Age group: 40-49	0.539	0.456	0.635	0.445	0.377	0.526	0.446	0.377	0.526	0.455	0.385	0.536	0.456	0.386	0.538
Age group: 60-69	1.288	1.156	1.435	1.804	1.619	2.012	1.813	1.626	2.021	1.687	1.515	1.879	1.687	1.515	1.879
Age group: 70-79	1.165	1.049	1.294	2.058	1.854	2.287	2.076	1.869	2.307	1.845	1.661	2.050	1.846	1.663	2.051
Age group: 80-89	0.883	0.793	0.985	2.991	2.679	3.340	3.034	2.718	3.389	3.611	3.241	4.026	3.651	3.276	4.070
Age group: 90+	0.356	0.302	0.419	10.399	8.814	12.251	10.689	9.060	12.591	7.954	6.748	9.361	8.079	6.855	9.507
Gendermale	1.044	1.022	1.066	1.360	1.330	1.390	1.356	1.326	1.385	1.536	1.502	1.570	1.530	1.497	1.564
LanguageNL	2.460	2.398	2.524	1.512	1.473	1.551	1.246	1.214	1.278	1.662	1.620	1.706	1.336	1.302	1.371
Reason: Dementia	0.198	0.176	0.222	0.198	0.175	0.222	0.198	0.176	0.222	0.197	0.175	0.221	0.198	0.175	0.222
Reason: Multimorbidity	0.305	0.296	0.315	0.303	0.294	0.313	0.303	0.294	0.313	0.302	0.293	0.312	0.303	0.293	0.312
Reason: Nervous system diseases	0.179	0.172	0.187	0.178	0.171	0.186	0.178	0.171	0.186	0.178	0.171	0.186	0.178	0.171	0.186
Reason: Others	0.104	0.093	0.115	0.103	0.092	0.114	0.103	0.093	0.114	0.103	0.092	0.113	0.103	0.092	0.114
Reason: Psychiatric disorders	0.377	0.340	0.418	0.393	0.354	0.435	0.393	0.354	0.435	0.392	0.353	0.434	0.392	0.353	0.435
Reason: Specific diseases	0.190	0.182	0.198	0.188	0.181	0.196	0.189	0.181	0.196	0.188	0.181	0.196	0.188	0.181	0.196
Basis: advanced	0.196	0.178	0.215	0.198	0.180	0.217	0.198	0.180	0.217	0.197	0.179	0.217	0.198	0.179	0.217
Suffering: both	2.024	1.972	2.077	2.023	1.972	2.077	2.024	1.973	2.078	2.024	1.972	2.077	2.024	1.973	2.078
Suffering: mental	0.629	0.586	0.674	0.622	0.580	0.667	0.622	0.580	0.667	0.626	0.583	0.671	0.626	0.583	0.671
Term: Short term	1.892	1.830	1.957	1.902	1.839	1.967	1.903	1.840	1.968	1.904	1.841	1.969	1.904	1.841	1.969
Place: Hospital	0.822	0.803	0.842	0.821	0.802	0.841	0.821	0.802	0.841	0.823	0.804	0.843	0.823	0.804	0.843
Place: Nursing home	0.461	0.446	0.477	0.458	0.442	0.474	0.458	0.442	0.474	0.458	0.443	0.474	0.458	0.443	0.474
Place: Other	0.132	0.121	0.143	0.130	0.119	0.140	0.130	0.119	0.140	0.130	0.119	0.140	0.130	0.119	0.140

© 2025 Wels J and Hamarat N. *JAMA Network Open*.

Place: Palliative care	0.190	0.177	0.204	0.189	0.176	0.203	0.189	0.176	0.203	0.189	0.176	0.203	0.189	0.175	0.203
Age group: 15-29 * Year	0.989	0.956	1.025	0.997	0.964	1.033	0.997	0.963	1.033	0.990	0.957	1.026	0.991	0.957	1.026
Age group: 30-39 * Year	0.993	0.975	1.011	1.000	0.982	1.018	1.000	0.982	1.019	0.991	0.973	1.010	0.992	0.974	1.010
Age group: 40-49 * Year	0.992	0.980	1.003	1.006	0.994	1.018	1.007	0.995	1.019	0.992	0.980	1.004	0.992	0.980	1.003
Age group: 60-69 * Year	1.018	1.011	1.026	1.009	1.001	1.016	1.008	1.000	1.015	1.018	1.011	1.026	1.018	1.011	1.026
Age group: 70-79 * Year	1.035	1.028	1.043	1.034	1.027	1.041	1.033	1.025	1.040	1.035	1.028	1.042	1.035	1.028	1.042
Age group: 80-89 * Year	1.052	1.044	1.059	1.046	1.038	1.054	1.045	1.037	1.052	1.051	1.043	1.059	1.051	1.044	1.059
Age group: 90+ * Year	1.059	1.049	1.070	1.019	1.008	1.029	1.017	1.007	1.028	1.063	1.052	1.074	1.064	1.053	1.075

© 2025 Wels J and Hamarat N. *JAMA Network Open*.

Model 8. Interaction between year and gender

	Incidence rate ratio			Prevalence ratio Includng Brussels			1. excluding Brussels			Prevalence ratio (sensitivity check) 2. baseline values, including Brussels			3. baseline values, excluding Brussels		
	RR	2.5 %	97.5 %	PR	2.5 %	97.5 %	PR	2.5 %	97.5 %	PR	2.5 %	97.5 %	PR	2.5 %	97.5 %
(Intercept)	0.589	0.548	0.634	0.000	0.000	0.000	0.000	0.000	0.000	0.000	0.000	0.000	0.000	0.000	0.000
year	1.073	1.070	1.076	1.057	1.054	1.060	1.057	1.054	1.060	1.071	1.068	1.074	1.071	1.068	1.074
Gender: male	1.150	1.079	1.226	1.508	1.414	1.608	1.500	1.407	1.599	1.565	1.468	1.669	1.562	1.465	1.666
Age group: 15-29	0.212	0.176	0.253	0.165	0.137	0.197	0.168	0.139	0.200	0.142	0.118	0.170	0.144	0.120	0.172
Age group: 30-39	0.292	0.263	0.323	0.312	0.281	0.346	0.320	0.288	0.355	0.247	0.223	0.274	0.251	0.226	0.278
Age group: 40-49	0.486	0.455	0.520	0.489	0.457	0.523	0.495	0.463	0.529	0.411	0.385	0.440	0.412	0.386	0.441
Age group: 60-69	1.631	1.566	1.700	1.999	1.919	2.083	1.982	1.903	2.066	2.147	2.061	2.237	2.148	2.062	2.239
Age group: 70-79	1.878	1.805	1.954	3.268	3.141	3.400	3.238	3.113	3.370	2.964	2.850	3.085	2.965	2.851	3.086
Age group: 80-89	1.798	1.728	1.872	5.659	5.438	5.891	5.633	5.412	5.864	7.294	7.008	7.594	7.400	7.109	7.704
Age group: 90+	0.837	0.797	0.879	13.172	12.534	13.842	13.241	12.600	13.915	19.656	18.703	20.657	20.216	19.236	21.246
LanguageNL	2.451	2.389	2.515	1.512	1.473	1.551	1.245	1.214	1.278	1.656	1.614	1.699	1.332	1.298	1.366
Reason: Dementia	0.199	0.177	0.224	0.199	0.176	0.223	0.199	0.177	0.223	0.198	0.176	0.222	0.198	0.176	0.222
Reason: Multimorbidity	0.306	0.297	0.316	0.304	0.294	0.314	0.304	0.294	0.314	0.304	0.294	0.313	0.304	0.294	0.314
Reason: Nervous system diseases	0.180	0.173	0.187	0.179	0.172	0.186	0.179	0.172	0.186	0.179	0.172	0.186	0.179	0.172	0.186
Reason: Others	0.104	0.094	0.115	0.103	0.093	0.114	0.103	0.093	0.114	0.103	0.093	0.114	0.103	0.093	0.114
Reason: Psychiatric disorders	0.372	0.335	0.412	0.388	0.349	0.429	0.388	0.349	0.429	0.387	0.349	0.429	0.388	0.349	0.430
Reason: Specific diseases	0.190	0.183	0.198	0.189	0.181	0.196	0.189	0.181	0.196	0.188	0.181	0.196	0.189	0.181	0.196
Basis: advanced	0.200	0.182	0.220	0.200	0.182	0.220	0.200	0.182	0.220	0.201	0.182	0.220	0.201	0.183	0.221
Suffering: both	2.014	1.963	2.067	2.016	1.965	2.069	2.017	1.966	2.071	2.013	1.961	2.066	2.013	1.962	2.066
Suffering: mental	0.630	0.587	0.676	0.624	0.582	0.669	0.625	0.582	0.670	0.626	0.583	0.671	0.626	0.583	0.671
Term: Short term	1.897	1.834	1.962	1.905	1.842	1.971	1.906	1.843	1.972	1.909	1.846	1.974	1.909	1.846	1.975
Place: Hospital	0.823	0.803	0.842	0.822	0.802	0.841	0.822	0.802	0.842	0.824	0.804	0.844	0.824	0.804	0.843
Place: Nursing home	0.462	0.446	0.478	0.458	0.442	0.473	0.458	0.442	0.474	0.458	0.442	0.474	0.458	0.442	0.474
Place: Other	0.132	0.122	0.143	0.130	0.120	0.141	0.130	0.120	0.141	0.130	0.120	0.141	0.130	0.120	0.141

© 2025 Wels J and Hamarat N. *JAMA Network Open*.

Place: Palliative care	0.192	0.178	0.206	0.190	0.177	0.205	0.190	0.177	0.205	0.191	0.177	0.205	0.191	0.177	0.205
Gender: male. * Year	0.994	0.989	0.998	0.993	0.989	0.997	0.993	0.989	0.997	0.999	0.995	1.003	0.999	0.995	1.003

© 2025 Wels J and Hamarat N. *JAMA Network Open*.

Model 9. Interaction between year and language/region

	Incidence rate ratio			Prevalence ratio<br>Includng Brussels			Prevalence ratio (sensitivity check)<br>1. excluding Brussels			2. baseline values, including Brussels			3. baseline values, excluding Brussels		
	RR	2.5 %	97.5 %	PR	2.5 %	97.5 %	PR	2.5 %	97.5 %	PR	2.5 %	97.5 %	PR	2.5 %	97.5 %
(Intercept)	0.542	0.494	0.594	0.000	0.000	0.000	0.000	0.000	0.000	0.000	0.000	0.000	0.000	0.000	0.000
year	1.079	1.074	1.084	1.068	1.063	1.073	1.067	1.062	1.072	1.079	1.074	1.084	1.080	1.075	1.085
LanguageNL	2.884	2.655	3.136	1.961	1.805	2.132	1.593	1.466	1.733	1.938	1.783	2.108	1.569	1.444	1.706
Age group: 15-29	0.212	0.176	0.253	0.165	0.137	0.197	0.168	0.139	0.200	0.142	0.118	0.170	0.144	0.120	0.172
Age group: 30-39	0.292	0.262	0.323	0.312	0.281	0.345	0.320	0.288	0.354	0.247	0.222	0.274	0.251	0.226	0.278
Age group: 40-49	0.486	0.454	0.519	0.488	0.456	0.521	0.494	0.462	0.528	0.411	0.384	0.439	0.412	0.385	0.440
Age group: 60-69	1.633	1.567	1.701	2.000	1.919	2.084	1.982	1.903	2.065	2.148	2.062	2.238	2.149	2.063	2.240
Age group: 70-79	1.877	1.805	1.954	3.263	3.137	3.396	3.233	3.108	3.364	2.964	2.849	3.084	2.965	2.850	3.085
Age group: 80-89	1.798	1.727	1.871	5.658	5.437	5.890	5.630	5.409	5.860	7.292	7.006	7.592	7.397	7.107	7.701
Age group: 90+	0.836	0.796	0.878	13.187	12.548	13.857	13.253	12.612	13.927	19.644	18.692	20.644	20.207	19.227	21.236
Gender: male	1.046	1.023	1.068	1.363	1.334	1.393	1.359	1.329	1.389	1.538	1.505	1.572	1.532	1.499	1.566
Reason: Dementia	0.199	0.177	0.223	0.198	0.176	0.222	0.198	0.176	0.223	0.198	0.176	0.222	0.198	0.176	0.222
Reason: Multimorbidity	0.306	0.296	0.316	0.304	0.294	0.313	0.304	0.294	0.314	0.303	0.294	0.313	0.304	0.294	0.313
Reason: Nervous system diseases	0.180	0.172	0.187	0.179	0.171	0.186	0.179	0.171	0.186	0.179	0.171	0.186	0.179	0.172	0.186
Reason: Others	0.104	0.094	0.116	0.104	0.093	0.115	0.104	0.093	0.115	0.103	0.093	0.114	0.103	0.093	0.114
Reason: Psychiatric disorders	0.373	0.336	0.413	0.388	0.350	0.430	0.388	0.350	0.430	0.387	0.349	0.429	0.388	0.349	0.430
Reason: Specific diseases	0.190	0.183	0.198	0.188	0.181	0.196	0.189	0.181	0.196	0.188	0.181	0.196	0.188	0.181	0.196
Basis: advanced	0.199	0.181	0.219	0.199	0.181	0.219	0.199	0.181	0.219	0.200	0.182	0.220	0.200	0.182	0.220
Suffering: both	2.015	1.963	2.068	2.017	1.966	2.070	2.018	1.967	2.071	2.014	1.962	2.067	2.014	1.962	2.067
Suffering: mental	0.628	0.586	0.674	0.622	0.580	0.667	0.623	0.580	0.668	0.625	0.582	0.670	0.625	0.582	0.670
Term: Short term	1.898	1.835	1.963	1.907	1.844	1.973	1.908	1.845	1.974	1.910	1.847	1.976	1.910	1.847	1.976
Place: Hospital	0.823	0.804	0.843	0.822	0.803	0.842	0.822	0.803	0.842	0.824	0.805	0.844	0.824	0.805	0.844
Place: Nursing home	0.461	0.446	0.477	0.457	0.442	0.473	0.457	0.442	0.473	0.458	0.442	0.474	0.458	0.442	0.474
Place: Other	0.132	0.122	0.143	0.130	0.120	0.141	0.130	0.120	0.141	0.130	0.120	0.141	0.130	0.120	0.141

© 2025 Wels J and Hamarat N. *JAMA Network Open*.

Place: Palliative care	0.192	0.178	0.206	0.190	0.176	0.204	0.190	0.176	0.204	0.190	0.177	0.204	0.190	0.177	0.204
year:LanguageNL	0.989	0.984	0.994	0.983	0.978	0.988	0.984	0.979	0.989	0.990	0.985	0.995	0.989	0.984	0.994

© 2025 Wels J and Hamarat N. *JAMA Network Open*.

eFigure 1. Marginal Effects in the Fully Adjusted Model

Model 1. Marginal effects of year

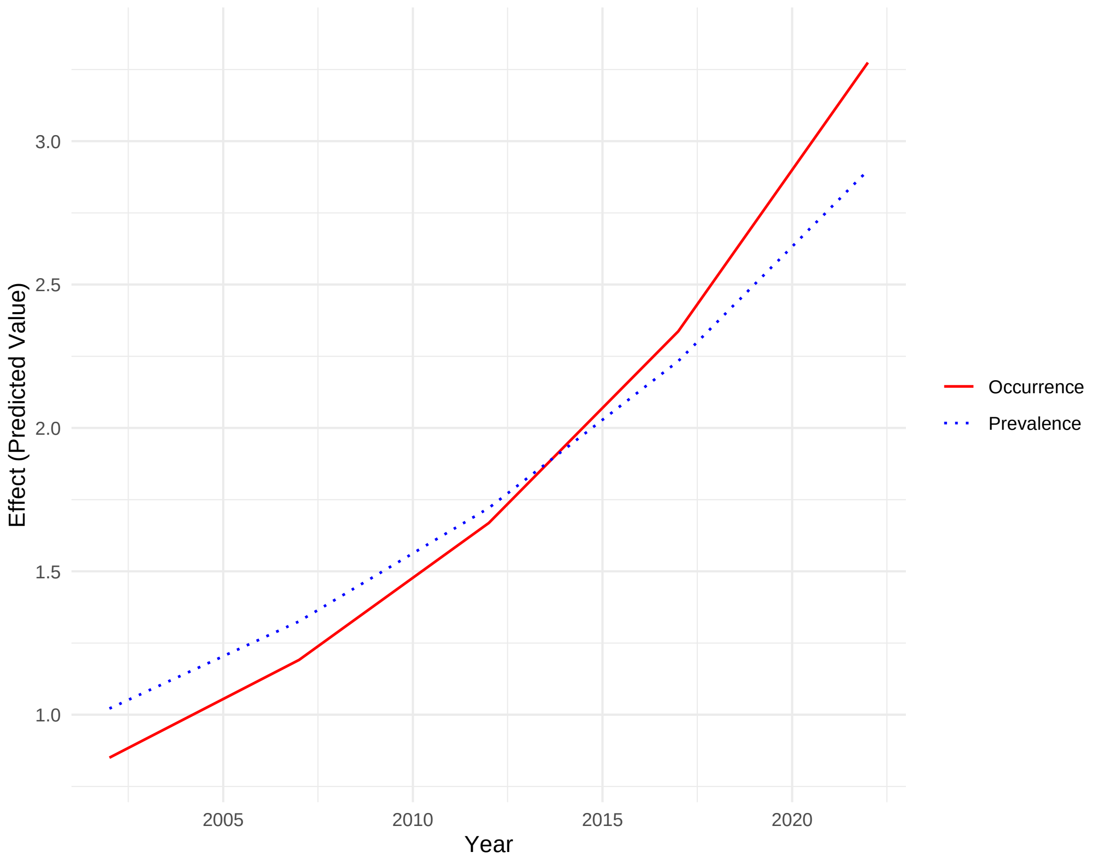

© 2025 Wels J and Hamarat N. *JAMA Network Open*.

Model 2. Marginal effects of the interaction between age group and year

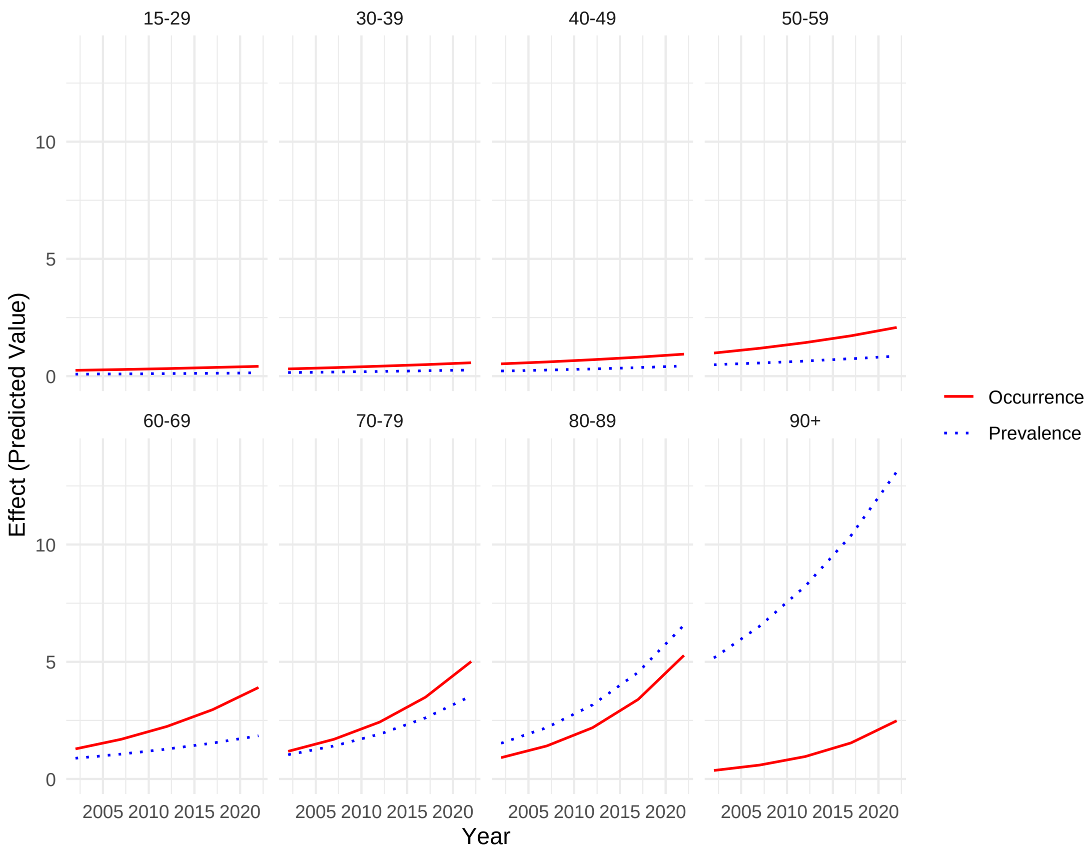

© 2025 Wels J and Hamarat N. *JAMA Network Open*.

Model 3. Marginal effects of the interaction between gender and year

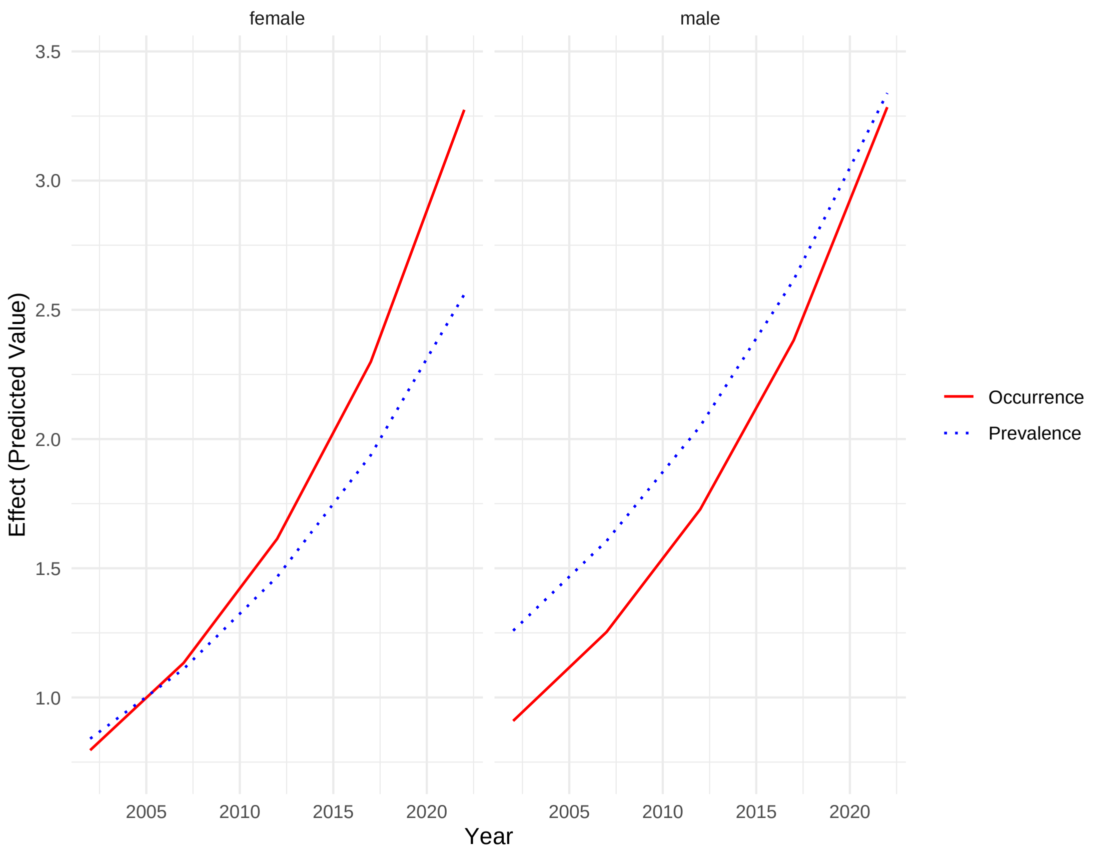

© 2025 Wels J and Hamarat N. *JAMA Network Open*.

Model 4. Marginal effects of the interaction between language/region and year

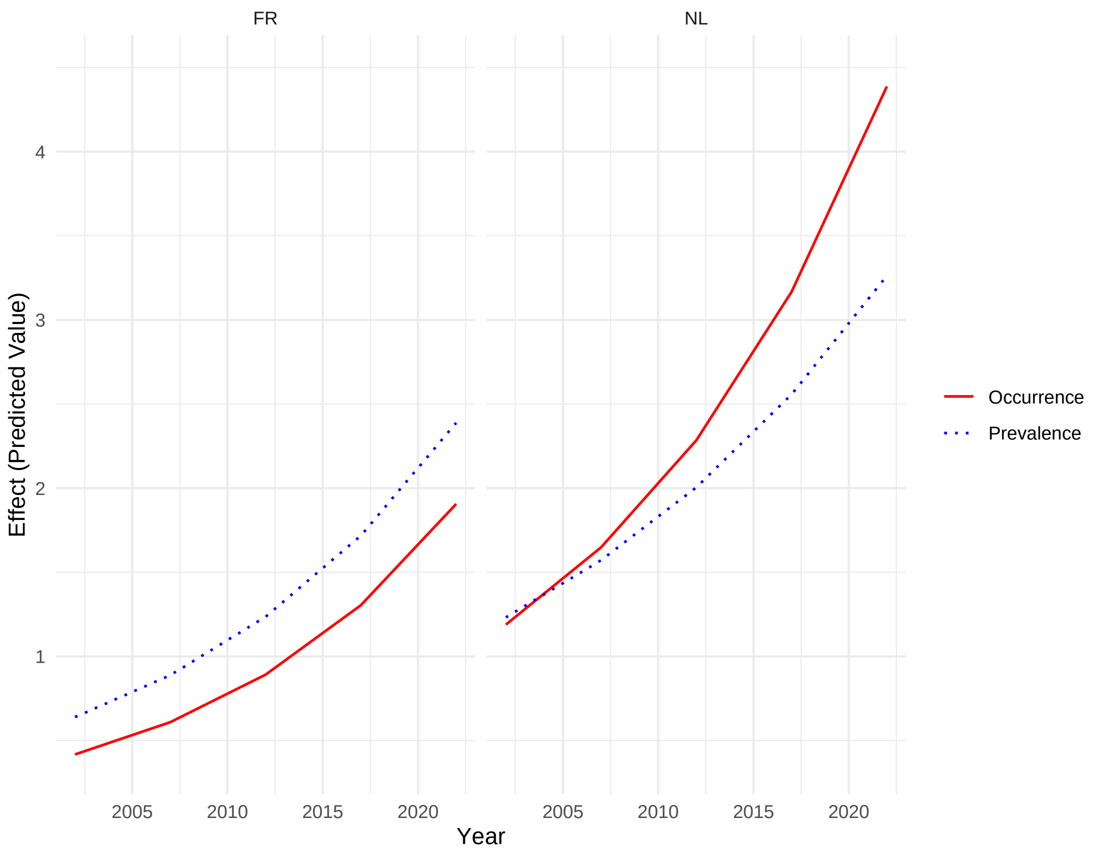

© 2025 Wels J and Hamarat N. *JAMA Network Open*.

Model 5. Marginal effects of the interaction between reason for euthanasia and year

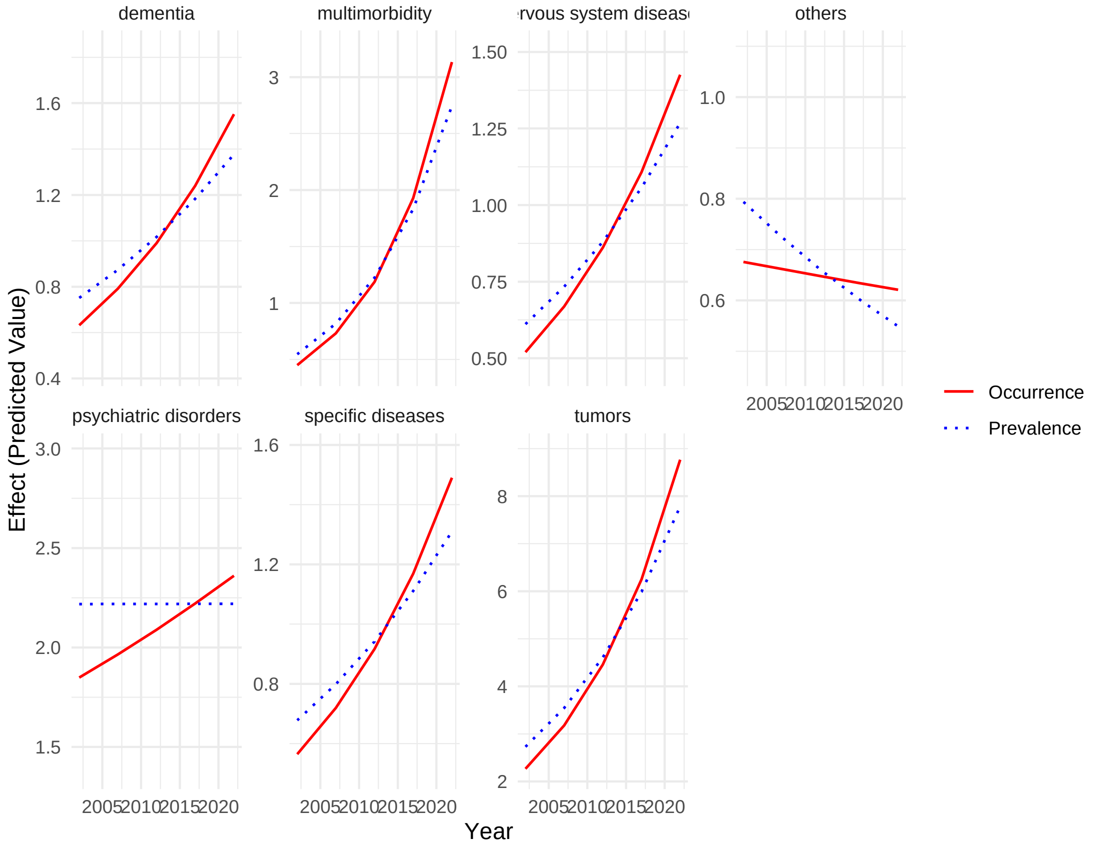

© 2025 Wels J and Hamarat N. *JAMA Network Open*.

Model 6. Marginal effects of the interaction between basis of euthanasia and year

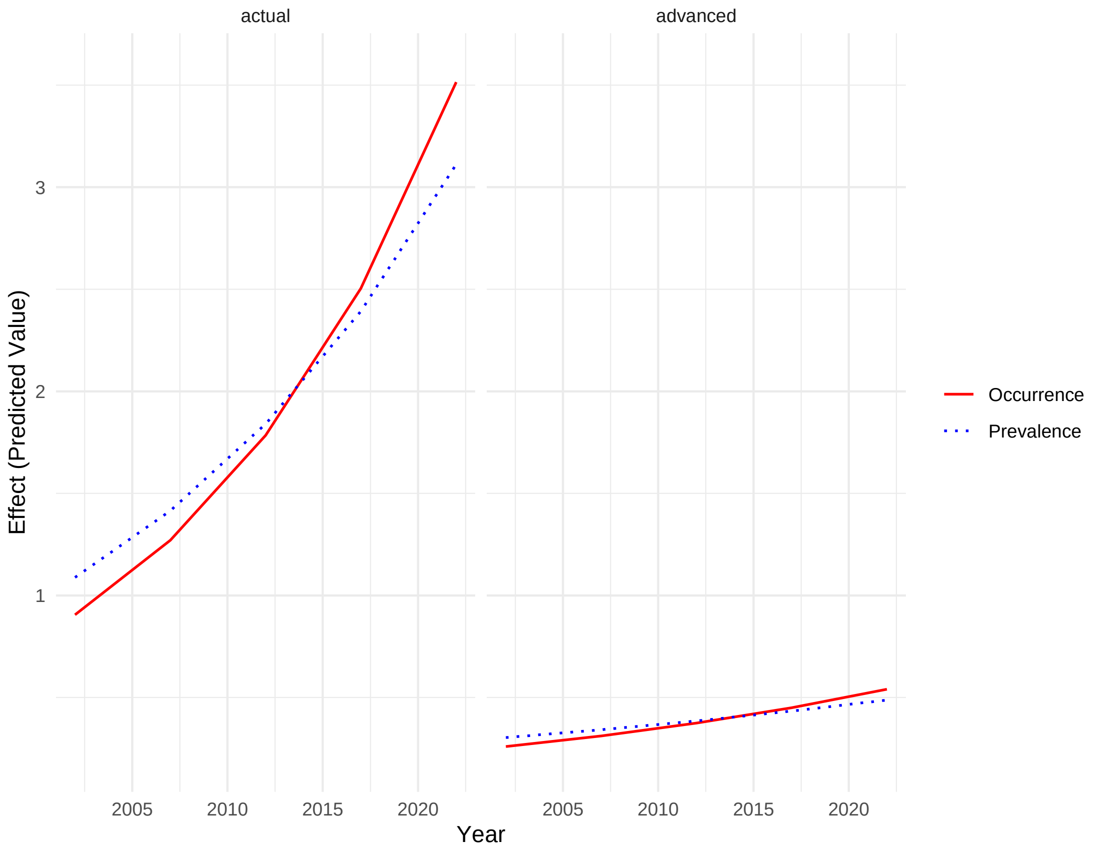

© 2025 Wels J and Hamarat N. *JAMA Network Open*.

Model 7. Marginal effects of the interaction between type of suffering and year

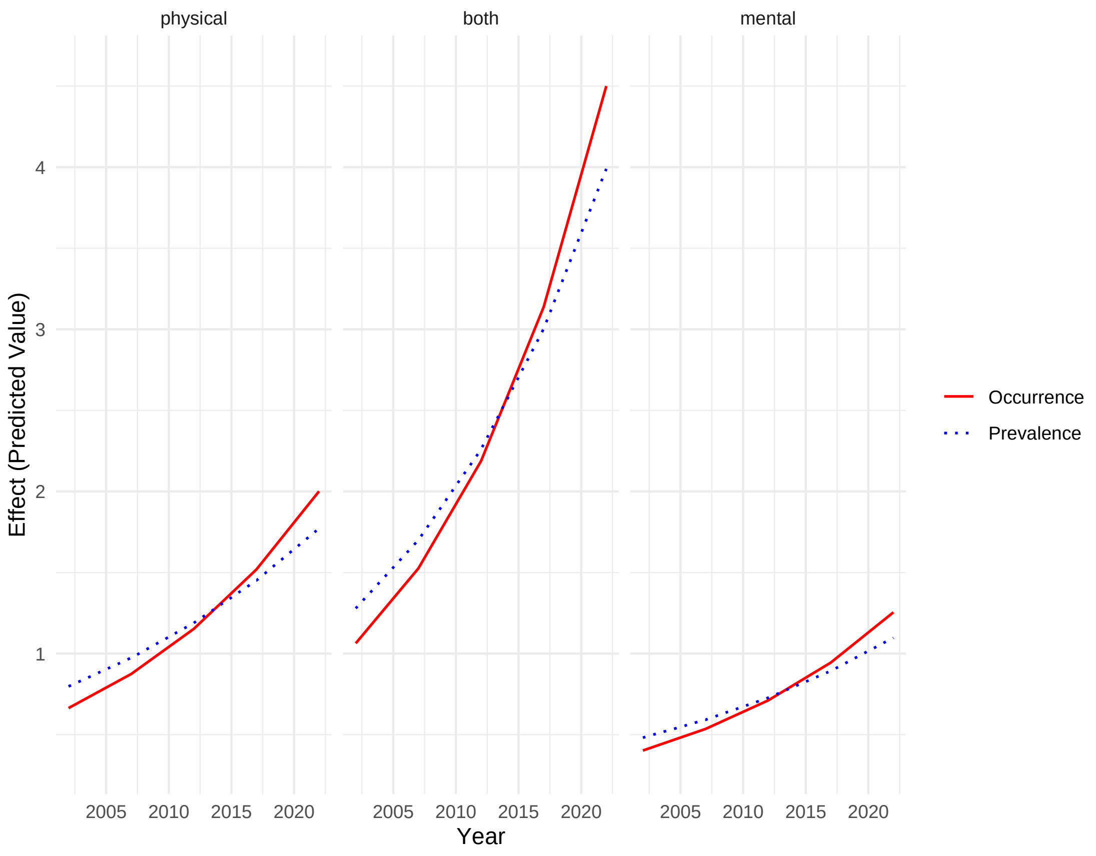

© 2025 Wels J and Hamarat N. *JAMA Network Open*.

Model 8. Marginal effects of the interaction between term of death and year

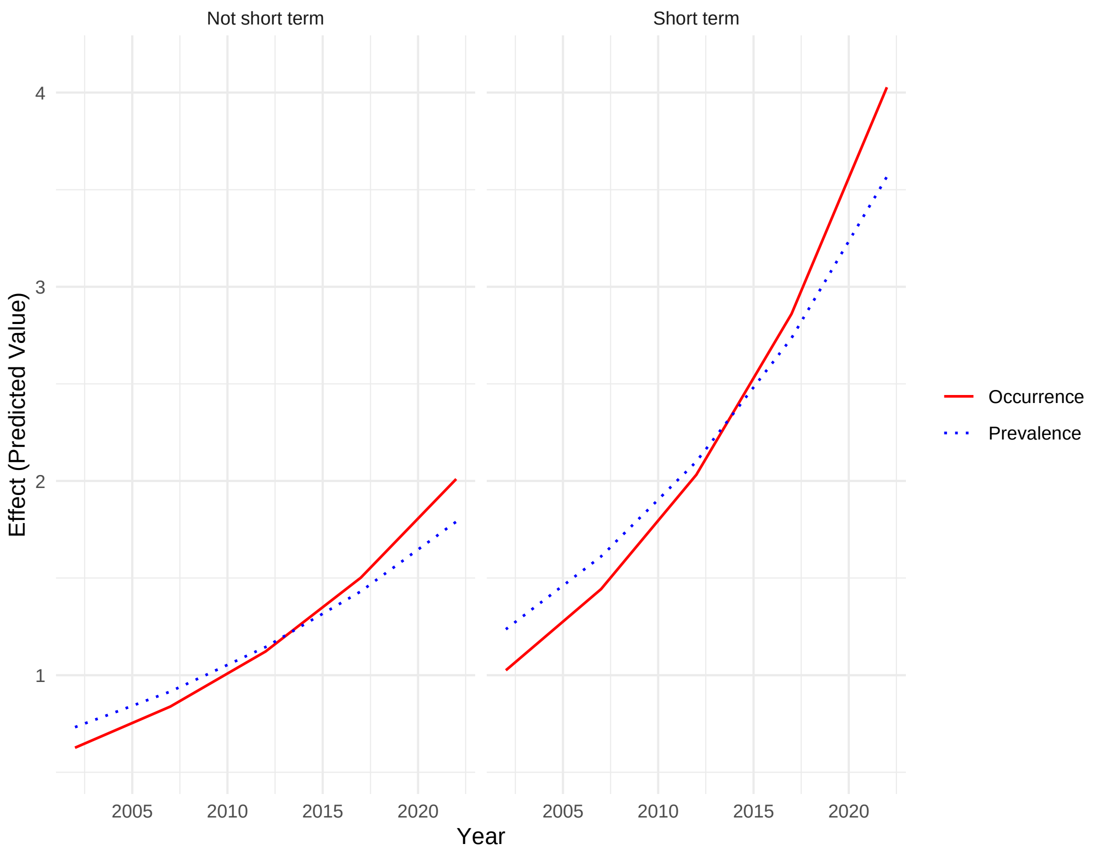

© 2025 Wels J and Hamarat N. *JAMA Network Open*.

Model 9. Marginal effects of the interaction between place of death and year

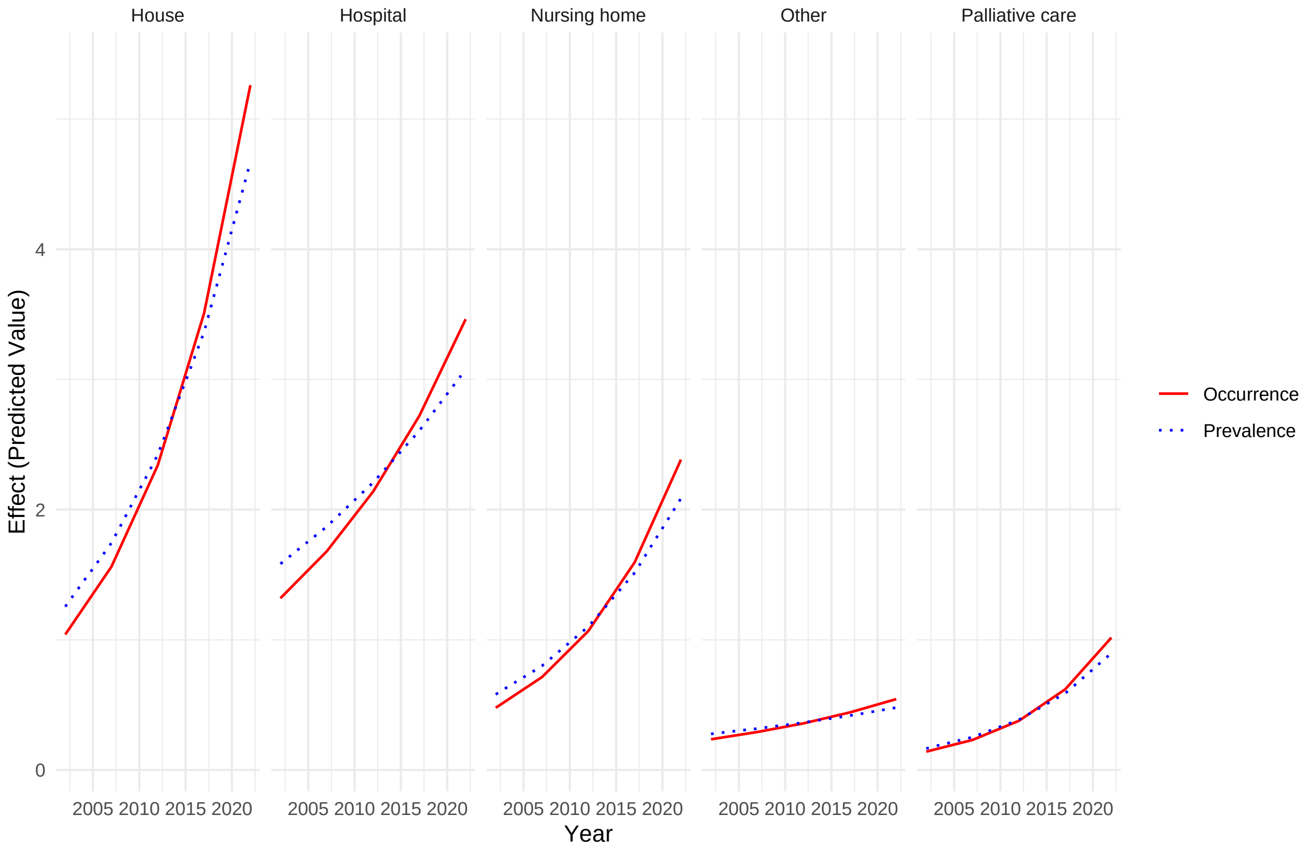

© 2025 Wels J and Hamarat N. *JAMA Network Open*.

eFigure 2. Marginal Effects (Year as Categorical)

Model 1. Marginal effects of year

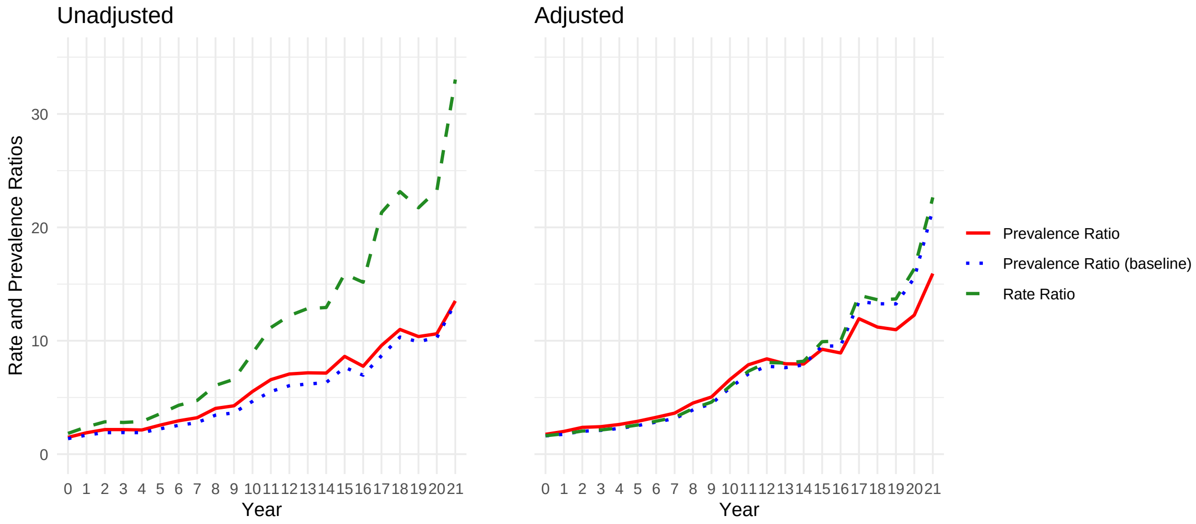

© 2025 Wels J and Hamarat N. *JAMA Network Open*.

Model 2. Marginal effects of the interaction between age group and year

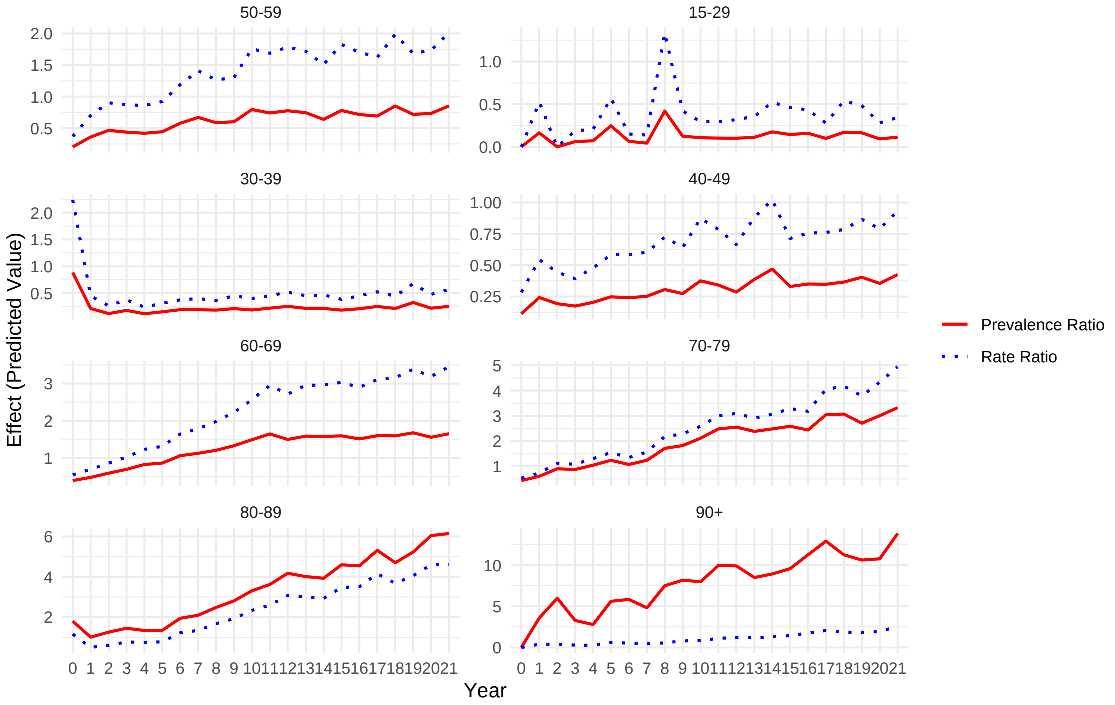

© 2025 Wels J and Hamarat N. *JAMA Network Open*.

Model 3. Marginal effects of the interaction between gender and year

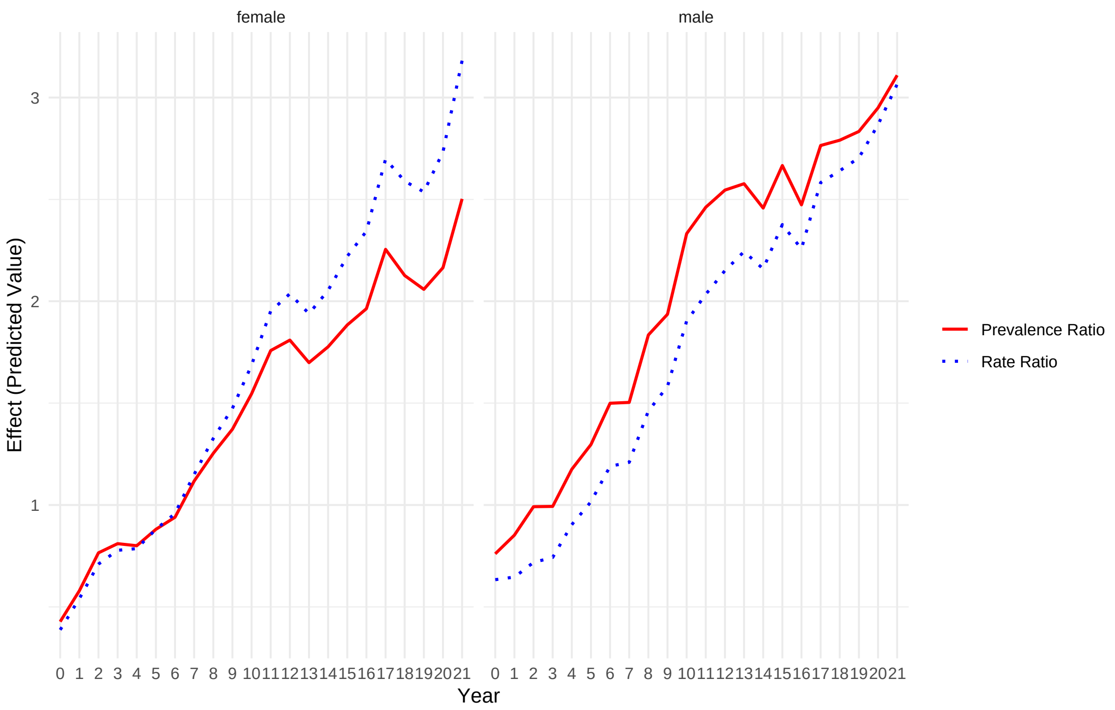

© 2025 Wels J and Hamarat N. *JAMA Network Open*.

Model 4. Marginal effects of the interaction between language/region and year

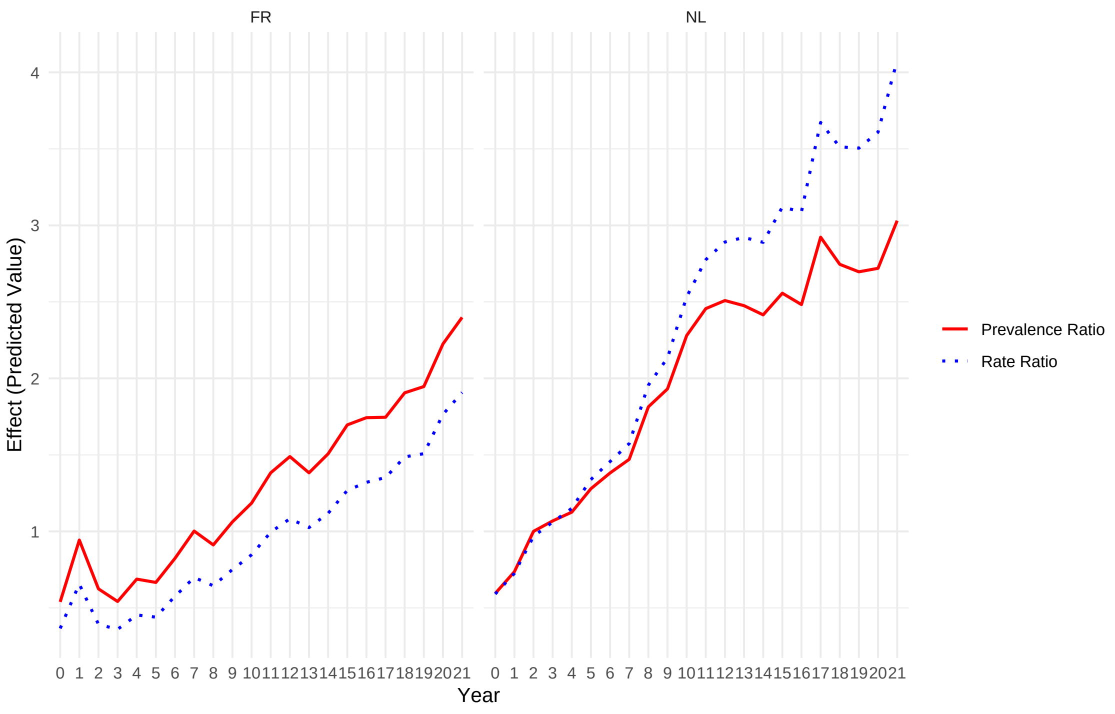

© 2025 Wels J and Hamarat N. *JAMA Network Open*.

Model 5. Marginal effects of the interaction between reason for euthanasia and year

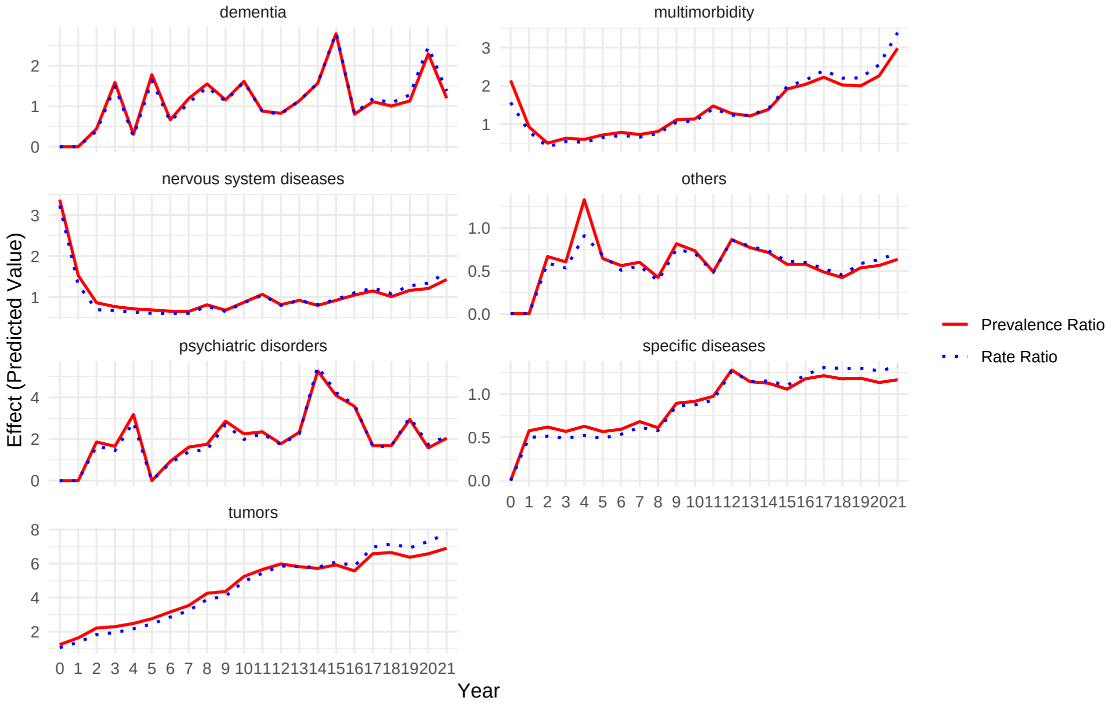

© 2025 Wels J and Hamarat N. *JAMA Network Open*.

Model 6. Marginal effects of the interaction between basis of euthanasia and year

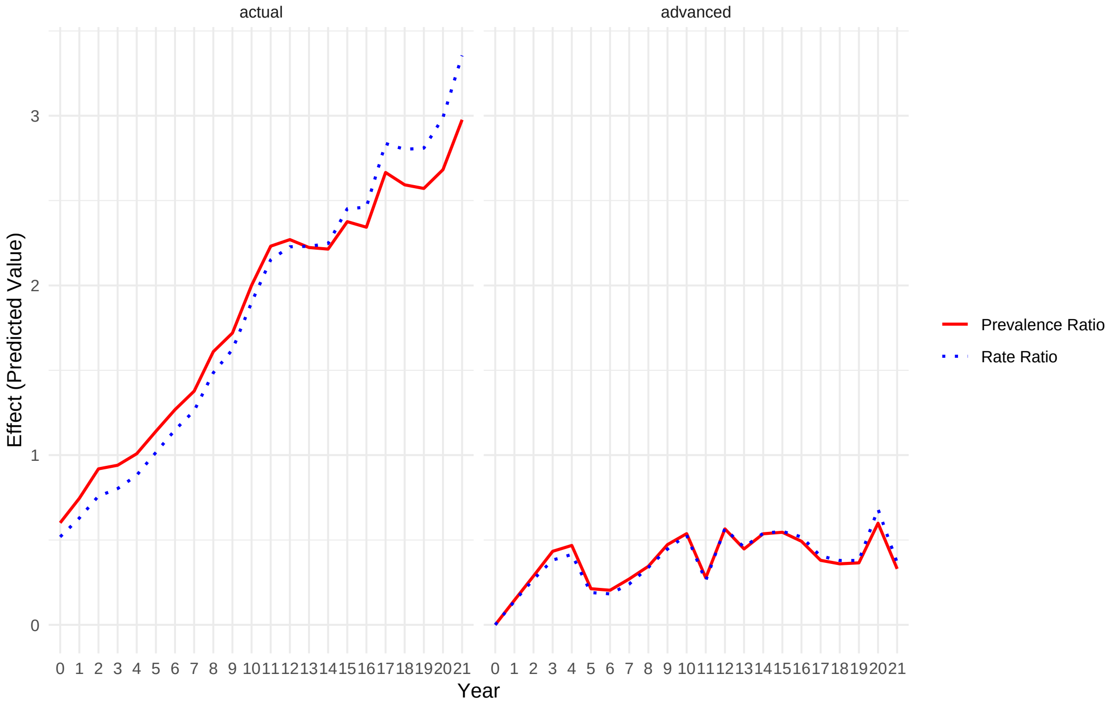

© 2025 Wels J and Hamarat N. *JAMA Network Open*.

Model 7. Marginal effects of the interaction between type of suffering and year

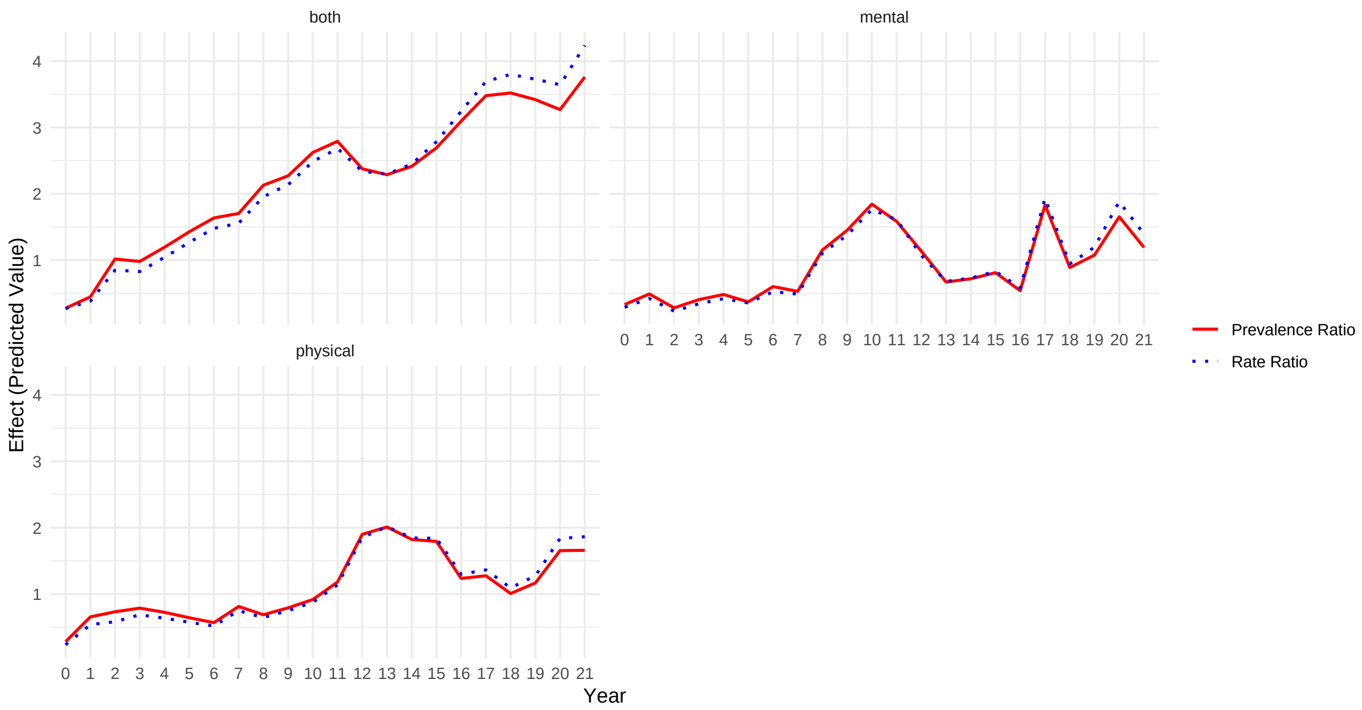

© 2025 Wels J and Hamarat N. *JAMA Network Open*.

Model 8. Marginal effects of the interaction between term of death and year

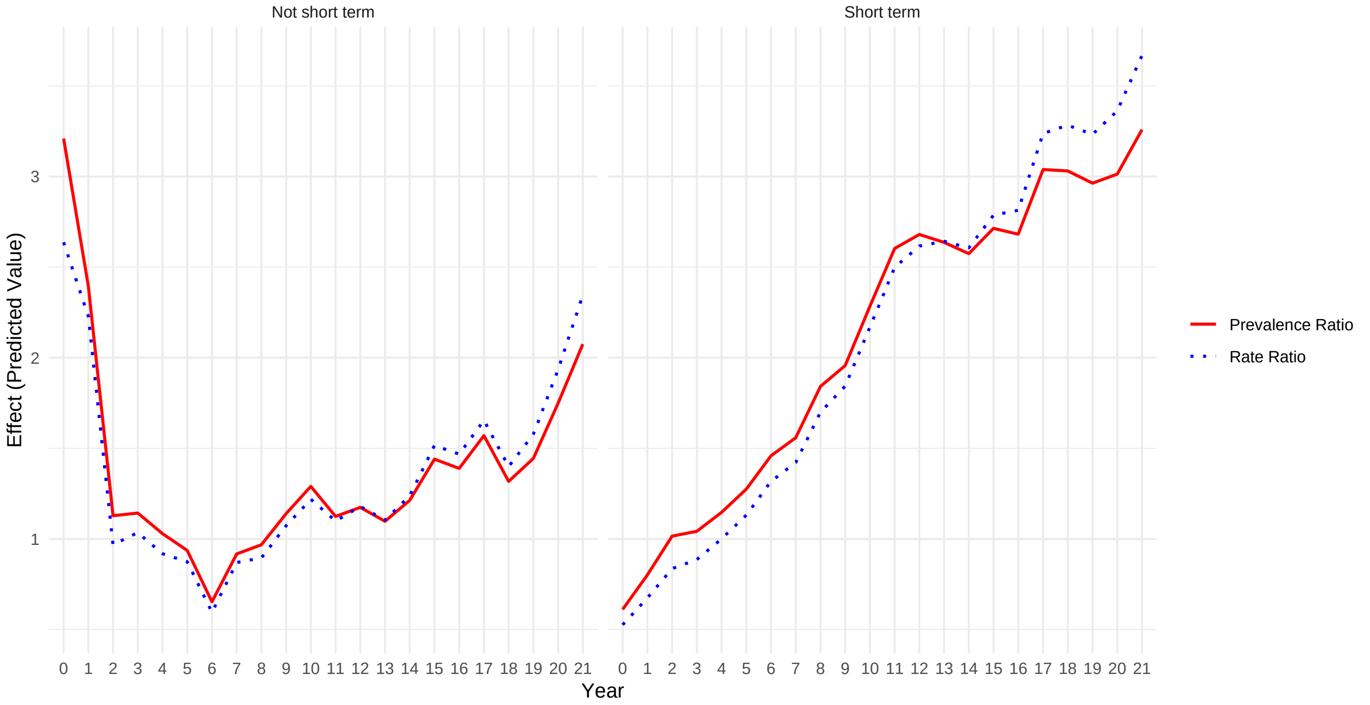

© 2025 Wels J and Hamarat N. *JAMA Network Open*.

Model 9. Marginal effects of the interaction between place of death and year

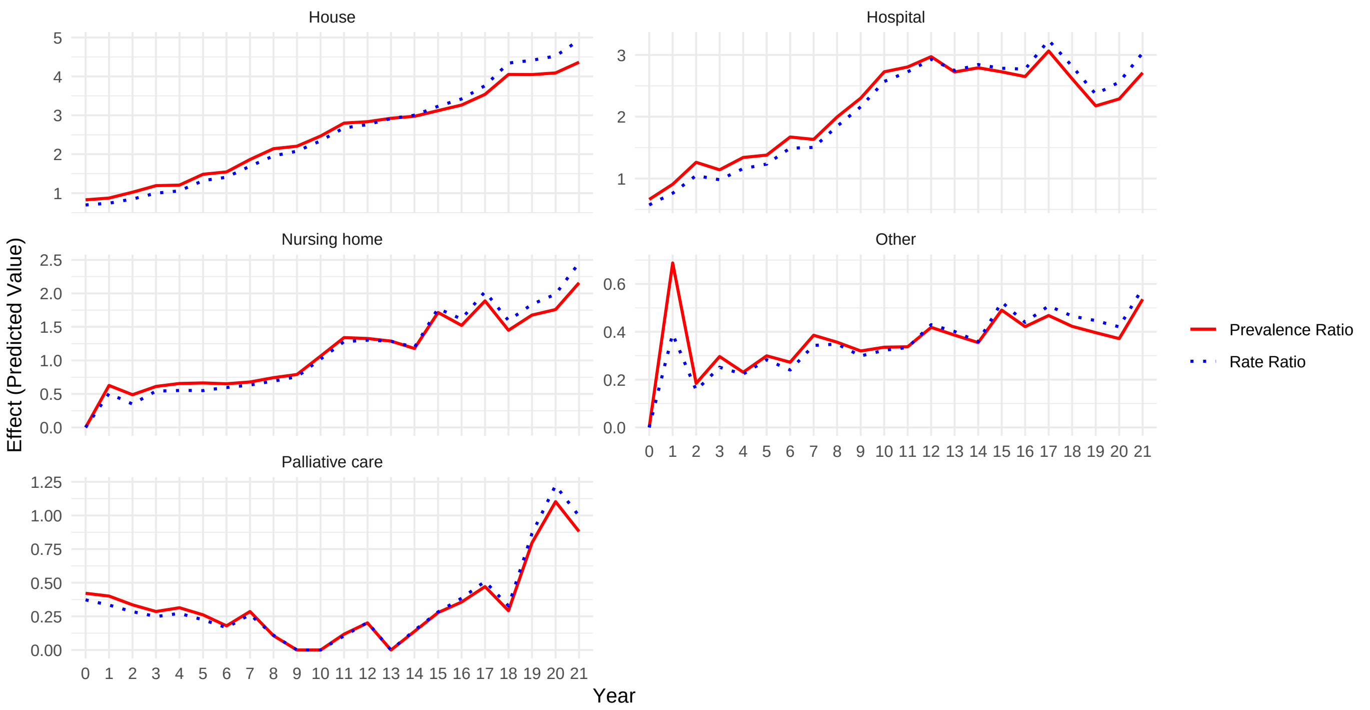

© 2025 Wels J and Hamarat N. *JAMA Network Open*.

eTable 4. Linear Trends of Year by Time Period (2003-2015 and 2016-2022), Fully Adjusted Model Without Interaction

	2003-2015						2016-2022					
	Incidence rate ratio			Prevalence ratio			Incidence rate ratio			Prevalence ratio		
	RR	2.5 %	97.5 %	PR	2.5 %	97.5 %	RR	2.5 %	97.5 %	PR	2.5 %	97.5 %
(Intercept)	0.435	0.389	0.487	0.000	0.000	0.000	0.781	0.686	0.889	0.000	0.000	0.000
year	**1.118**	**1.112**	**1.124**	**1.100**	**1.094**	**1.106**	**1.048**	**1.042**	**1.055**	**1.033**	**1.027**	**1.039**
Age group: 15-29	0.198	0.148	0.259	0.147	0.110	0.193	0.227	0.178	0.284	0.182	0.142	0.228
Age group: 30-39	0.294	0.254	0.339	0.307	0.265	0.354	0.282	0.242	0.326	0.310	0.266	0.359
Age group: 40-49	0.488	0.444	0.536	0.455	0.414	0.500	0.471	0.427	0.518	0.508	0.461	0.559
Age group: 60-69	1.501	1.413	1.595	1.936	1.822	2.058	1.786	1.689	1.889	2.114	1.999	2.237
Age group: 70-79	1.564	1.474	1.659	2.776	2.618	2.946	2.191	2.077	2.313	3.779	3.582	3.990
Age group: 80-89	1.368	1.288	1.454	4.447	4.184	4.728	2.196	2.082	2.319	6.733	6.381	7.109
Age group: 90+	0.563	0.515	0.614	11.545	10.565	12.606	1.033	0.970	1.099	14.654	13.765	15.605
Gender: male	1.084	1.047	1.123	1.398	1.349	1.448	1.016	0.989	1.044	1.333	1.296	1.370
Language: NL	2.773	2.651	2.902	1.770	1.692	1.852	2.339	2.267	2.414	1.410	1.367	1.455
Reason: Dementia	0.222	0.181	0.270	0.220	0.179	0.267	0.196	0.169	0.226	0.197	0.170	0.226
Reason: Multimorbidity	0.246	0.232	0.262	0.242	0.227	0.257	0.331	0.318	0.343	0.330	0.317	0.342
Reason: Nervous system diseases	0.186	0.174	0.200	0.186	0.173	0.199	0.174	0.165	0.183	0.173	0.164	0.182
Reason: Others	0.151	0.127	0.177	0.147	0.124	0.173	0.088	0.077	0.100	0.087	0.076	0.099
Reason: Psychiatric disorders	0.368	0.314	0.429	0.384	0.328	0.448	0.364	0.316	0.418	0.382	0.332	0.438
Reason: Specific diseases	0.204	0.191	0.218	0.201	0.188	0.215	0.179	0.171	0.189	0.179	0.170	0.188
Basis: advanced	0.201	0.178	0.227	0.201	0.177	0.226	0.182	0.156	0.211	0.184	0.158	0.213
Suffering: both	1.835	1.761	1.912	1.837	1.763	1.914	2.179	2.107	2.253	2.177	2.105	2.252
Suffering: mental	0.649	0.585	0.719	0.645	0.581	0.714	0.597	0.542	0.657	0.587	0.532	0.645
Term: Short term	1.939	1.824	2.063	1.960	1.844	2.085	1.938	1.862	2.018	1.943	1.866	2.023
Place: Hospital	1.014	0.977	1.053	1.009	0.972	1.047	0.708	0.686	0.730	0.709	0.687	0.732

© 2025 Wels J and Hamarat N. *JAMA Network Open*.

Place: Nursing home	0.477	0.448	0.509	0.466	0.437	0.497	0.443	0.425	0.461	0.442	0.424	0.460
Place: Other	0.154	0.135	0.176	0.150	0.131	0.172	0.117	0.106	0.129	0.116	0.104	0.128
Place: Palliative care	0.188	0.145	0.239	0.187	0.144	0.237	0.192	0.178	0.207	0.191	0.177	0.206

© 2025 Wels J and Hamarat N. *JAMA Network Open*.

eFigure 3. Marginal Effects (Year as Categorical), Negative Binomial Regression

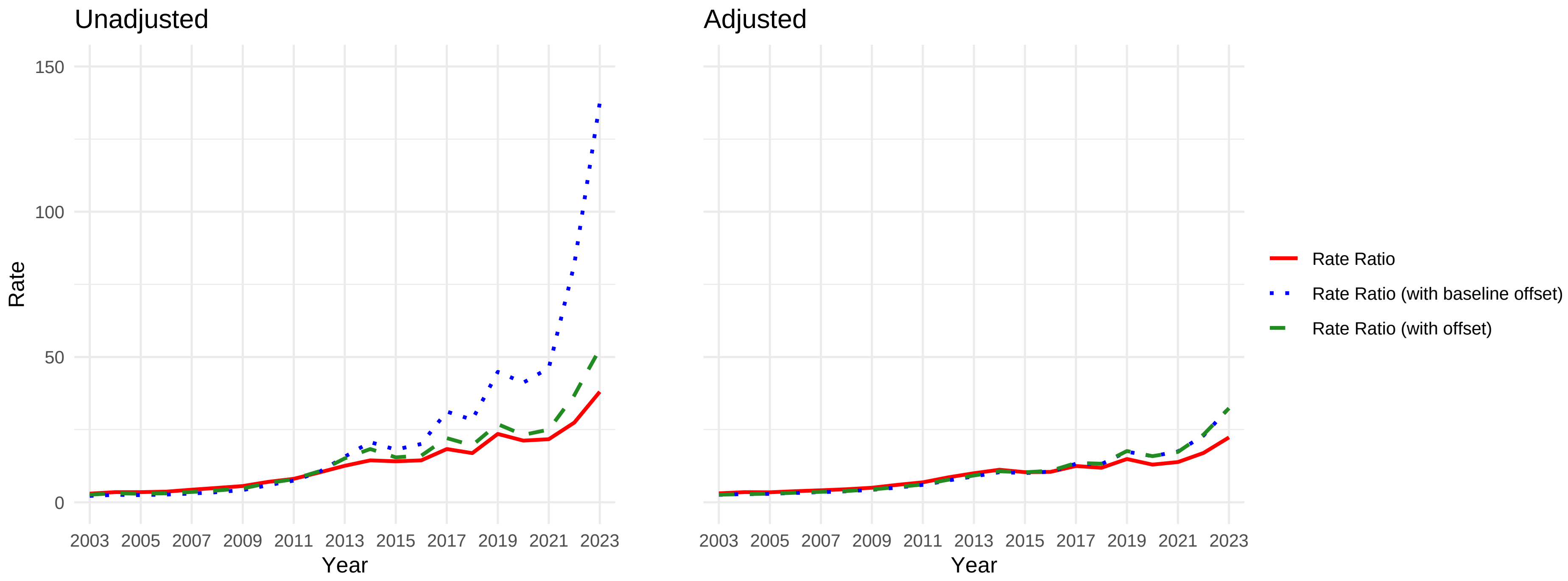

© 2025 Wels J and Hamarat N. *JAMA Network Open*.
