# Supplementary material for: Incidence and Prevalence of Reported Euthanasia Cases in Belgium, 2002 to 2023
Source: JAMA Netw Open. 2025 Apr 23;8(4):e256841. doi: 10.1001/jamanetworkopen.2025.6841 (PMC12019508; doi:10.1001/jamanetworkopen.2025.6841)
Supplement: Supplement 2. — Data Sharing Statement [file jamanetwopen-e256841-s002.pdf]

Data Sharing Statement

Wels. Incidence and Prevalence of Reported Euthanasia Cases in Belgium, 2002 to 2023. *JAMA Netw Open*. Published April 23, 2025. doi:10.1001/jamanetworkopen.2025.6841

Data
Data available: No

Additional Information
Explanation for why data not available: Access to data was granted by the Federal Commission for the Control and Evaluation of Euthanasia (FCCEE) on the 14th of May 2024. Data access is granted upon request to the FCCEE.
